# Serum EV-enriched proteomics reveals host-response subtypes and molecular similarity to organ dysfunction phenotypes in sepsis

**DOI:** 10.3389/fmed.2026.1896167

**Published:** 2026-10-07

**Authors:** Rui Ding, Shihan Zhang, Xinyan Liu, Haiming Jiang, Chunting Wang, Zhongheng Zhang, Xinhua Li, Xuan Song

**Affiliations:** 1 Yantai Affiliated Hospital of Binzhou Medical University, Yantai, China; 2 Department of Critical Care Medicine, Qilu Hospital, Shandong University, Jinan, China; 3 Department of Critical Care Medicine, Dong E Hospital, Liaocheng, China; 4 Department of Critical Care Medicine, Yantai Affiliated Hospital of Binzhou Medical University, Yantai, China; 5 Department of Critical Care Medicine, Shandong Provincial Hospital Affiliated to Shandong First Medical University, Jinan, China; 6 Department of Emergency Medicine, Sir Run Run Shaw Hospital, Zhejiang University School of Medicine, Hangzhou, China; 7 Provincial Key Laboratory of Precise Diagnosis and Treatment of Abdominal Infection, Sir Run Run Shaw Hospital, Zhejiang University School of Medicine, Hangzhou, China; 8 School of Medicine, Shaoxing University, Shaoxing, China; 9 Key Laboratory of Emergency and Trauma of Ministry of Education, Engineering Research Center for Hainan Biological Sample Resources of Major Diseases, The First Affiliated Hospital, Hainan Medical University, Haikou 571199, China; 10 Endocrine and Metabolic Diseases Hospital of Shandong First Medical University, Shandong Institute of Endocrine and Metabolic Diseases, Jinan Key Laboratory of Translational Medicine on Metabolic Diseases, Jinan, China; 11 Department of Critical Care Medicine, Public Health Clinical Center Affiliated to Shandong University, Shandong Public Health Clinical Center, Jinan, China

**Keywords:** EV-enriched proteomics, extracellular vesicles, molecular subtyping, organ dysfunction, sepsis

## Abstract

**Introduction:**

Sepsis is a heterogeneous syndrome characterized by dysregulated host responses and diverse patterns of organ dysfunction. This study aimed to characterize serum extracellular vesicle (EV)-enriched proteomic heterogeneity in sepsis and assess whether proteomic subtypes identified among Sepsis-only patients showed molecular similarity to established organ dysfunction phenotypes.

**Methods:**

In this single-center observational study, serum EV-enriched preparations were analyzed from 250 patients with sepsis and 50 healthy controls enrolled between June 2021 and December 2022. ARDS and AKI status was determined at blood sampling, and patients with neither condition were classified as Sepsis-only. After proteomic quality control, 231 patients and 48 controls were included. Non-negative matrix factorization was used to identify proteomic subtypes among Sepsis-only patients. Centroid projection and clinically adjusted sensitivity analyses evaluated cross-sectional molecular similarity between these subtypes and established S-AKI, S-ARDS, and S-ARDS&AKI phenotypes. An exploratory elastic-net protein panel was derived, and the downstream supervised workflow was evaluated using repeated nested cross-validation conditional on the original subtype labels.

**Results:**

A total of 4,754 protein groups were quantified across 279 samples. Three proteomic subtypes were identified among 96 Sepsis-only patients. S1 showed less pronounced inflammatory features, whereas S2 was characterized by cellular immune and metabolic-stress pathways and S3 by humoral immune, complement/coagulation, hemostatic, and acute-phase processes. These molecular patterns were not fully captured by conventional severity scores. The organ dysfunction groups showed partial and reference-dependent molecular similarity to S3 after clinical adjustment. A hypothesis-generating 15-protein panel captured features shared by S3 and established organ dysfunction relative to S2.

**Conclusions:**

Serum EV-enriched proteomics revealed distinct host-response subtypes within the Sepsis-only group and contemporaneous molecular features shared with established organ dysfunction. These findings advance the molecular characterization of sepsis heterogeneity and provide a focused framework for future longitudinal and external validation.

## 1 Introduction

Sepsis is life-threatening organ dysfunction caused by a dysregulated host response to infection (1–4). It imposes a substantial global burden, with an estimated 49 million new cases and 11 million deaths annually, accounting for nearly one-fifth of global mortality (5). Patients with sepsis exhibit a broad spectrum of clinical manifestations, ranging from mild symptoms to severe illness requiring mechanical ventilation, with complications such as multi-organ dysfunction, septic shock, and death. This clinical variability, combined with the absence of reliable predictive biomarkers, poses substantial challenges for effective triage and treatment (6). Improved early characterization of sepsis severity and host-response heterogeneity is therefore important for improving patient prognosis.

High-throughput omics technologies have accelerated precision medicine in infectious and critical care diseases (7). Transcriptomic and proteomic studies have contributed to biomarker discovery and molecular classification in sepsis (8, 9). However, limited sample sizes, heterogeneous study designs, and modest predictive performance have hindered broad clinical translation. Characterizing molecular profiles in clinically relevant sepsis cohorts may improve our understanding of host-response heterogeneity and support the development of more precise therapeutic strategies.

Extracellular vesicles (EVs) are lipid-bilayer-delimited particles released by cells and can carry proteins, lipids, and nucleic acids that reflect their cellular sources and extracellular environment (10, 11). In sepsis, Gong et al. reported that EV-associated Tenascin-C could exacerbate acute lung injury and intensify sepsis-associated inflammatory responses (12). In addition, Tu et al. reported that endothelial cell-derived EV-associated HSPA12B can downregulate NF-*κ*B pathway activation and regulate macrophage pro-inflammatory responses during sepsis (13). Accordingly, profiling a clinically accessible serum EV-enriched fraction may provide a complementary view of sepsis-related host-response heterogeneity (13, 14).

In this study, we analyzed serum EV-enriched proteomic profiles from patients with sepsis. We applied cluster analysis and identified three proteomic subtypes among patients without ARDS or AKI at blood sampling. We characterized the molecular and clinical features of these subtypes and examined their similarity to established organ dysfunction phenotypes. In addition, we derived an exploratory protein panel to provide a basis for future longitudinal studies evaluating the relationship between these molecular patterns and subsequent organ dysfunction.

## 2 Materials and methods

### 2.1 Study design and participants

This single-center observational study consecutively enrolled 250 patients with sepsis who were admitted to the ICU of Shandong Provincial Hospital Affiliated to Shandong First Medical University between June 2021 and December 2022. Patients were eligible if they were aged ≥18 years, met the Sepsis-3 criteria within 24 h of ICU admission, and had a serum sample collected immediately after diagnosis (1). The presence of ARDS and AKI at blood collection was assessed according to the Berlin definition (15) and KDIGO criteria (16), respectively. AKI assessment was based on serum creatinine and urine-output records, with renal imaging used as supportive information to evaluate structural abnormalities and possible causes of renal dysfunction. According to ARDS and AKI status at blood collection, patients were categorized into four mutually exclusive groups: Sepsis-only (*n* = 100), S-ARDS (*n* = 50), S-AKI (*n* = 50), and S-ARDS&AKI (*n* = 50). Subtype discovery was restricted to the Sepsis-only group to avoid established ARDS or AKI status influencing the cluster structure.

During the same study period, 50 healthy individuals undergoing routine physical examinations were enrolled as controls. These individuals had no clinical evidence of active infection or sepsis. The study was approved by the Ethics Committee of Shandong First Medical University (No. R202201170004), conducted in accordance with the Declaration of Helsinki, and written informed consent was obtained from all participants. This study was reported in accordance with the Strengthening the Reporting of Observational Studies in Epidemiology (STROBE) statement (17).

### 2.2 Serum sample collection and clinical data acquisition

For each participant, 2 mL of peripheral blood was obtained by venipuncture or from a pre-existing intravascular catheter. Samples were centrifuged at 3,000 × g for 15 min, and the serum was collected, aliquoted, and stored at −80 °C until EV enrichment and proteomic analysis.

Clinical data were obtained from electronic medical records. Baseline data included demographics, comorbidities, laboratory measurements, APACHE II and SOFA scores, and organ-support therapies recorded during the first 24 h after ICU admission. Organ-support variables represented treatment administered during the first ICU day and were not used to define ARDS or AKI status at blood collection. ICU length of stay and 28-day mortality were recorded as clinical outcomes.

### 2.3 Enrichment and characterization of serum EV-enriched preparations

Serum EV-enriched preparations were obtained using an EVtrap-based enrichment method (18). Briefly, EVtrap magnetic beads were added to serum samples according to the manufacturer's recommended ratio, followed by gentle shaking or vertical rotation for 1 h at room temperature to allow capture of EV-associated particles and material. The beads were collected using a magnetic separator and washed with PBS. The bead-bound EV-enriched material was then eluted by incubation with 100 mM triethylamine for 10 min and used for downstream characterization and proteomic analysis.

The morphology of particles in the EV-enriched preparations was examined by transmission electron microscopy (TEM; HT-7700, Hitachi, Japan). Briefly, EV-enriched suspensions were fixed with 2% paraformaldehyde and loaded onto Formvar-carbon-coated copper grids. The grids were washed with PBS, fixed with 1% glutaraldehyde for 5 min, and then washed with distilled water. After negative staining with uranyl oxalate, the grids were air-dried for 5–10 min and observed under TEM at an accelerating voltage of 80 kV. Representative images were acquired to evaluate vesicle-like structures in the EV-enriched preparations.

### 2.4 Nanoparticle tracking analysis

Nanocoulter nanoparticle analysis was performed using a RESUN-E03 system [Resun Intelligent Manufacturing (Shenzhen) Technology Co, Ltd, China] to determine the size distribution and particle concentration of serum EV-enriched preparations. Samples were diluted with sterile PBS to achieve an optimal detection concentration within the range of 10⁷–10⁹ particles/mL. Samples were gently mixed before measurement, and the acquired data were analyzed using the corresponding instrument software according to the manufacturer's instructions. The particle size distribution and particle concentration were calculated automatically. The results are presented as the mean particle size or median particle size with standard deviation, as appropriate.

### 2.5 Western blotting

Protein samples and molecular weight markers were loaded onto 12% SDS-PAGE gels. Electrophoresis was performed at 80 V until samples migrated through the stacking gel and then at 100 V until bromophenol blue reached the bottom of the gel. Proteins were transferred to polyvinylidene difluoride (PVDF) membranes. Membranes were blocked in 5% skimmed milk in TBST for 1 h and then incubated overnight at 4 °C with primary antibodies against TSG101 (1:1000, Abcam), Calnexin (1:1,000; Abcam), CD9 (1:1000, Yiwei Jianhua), and CD81 (1:1,000, Yiwei Jianhua). After washing, membranes were incubated with secondary antibodies diluted in 5% skimmed milk in TBST for 1 h at room temperature. Signals were developed using ECL reagents (K12049-D50, Advansta) and detected by Typhoon scanning.

### 2.6 Protein extraction and peptide enzymatic hydrolysis

Proteins were extracted from each sample using SDT lysis buffer (4% SDS, 100 mM Tris-HCl, pH 7.6). Protein concentrations were determined using the bicinchoninic acid assay (P0012, Beyotime). Loading buffer (5x) was added, and samples were boiled for 5 min before SDS-PAGE electrophoresis using a 4%–20% precast gradient gel at a constant voltage of 180 V for 45 min. Gels were stained with Coomassie Brilliant Blue R-250 (0,472–10G, AMRESCO). Equal amounts of protein from all samples were pooled to construct a spectral library.

All individual samples and pooled samples were digested with trypsin using the filter-aided sample preparation (FASP) method. Peptides from the pooled sample were fractionated using Thermo's high-pH reversed-phase peptide separation kit (Empore SPE Cartridges MCX, 30*μ*m, Waters). Fractionated pooled peptides and peptides from individual samples were desalted using C18 cartridges, lyophilized, and reconstituted in 0.1% formic acid (06,450, Fluka). Peptide concentrations were measured at OD280. Indexed retention time (iRT) standard peptides (Ki-3002–2, Biognosys) were added to pooled and individual peptide samples for data-dependent acquisition (DDA) and data-independent acquisition (DIA) mass spectrometry.

### 2.7 Mass spectrometry analysis

DDA analysis was performed using an Evosep One nanoscale flow chromatography system coupled to a timsTOF mass spectrometer. The following settings were used: positive ion detection; voltage, 1,500 V; ion source temperature, 180 °C; dry gas, 3 L/min; PASEF cycles, 8; TIMS accumulation time, 100 ms; TIMS range (ion mobility), 0.75–1.35 Vs/cm2; MS and MS/MS range, 100–1700Da; and dynamic exclusion time, 24 s. DIA analysis was performed using the same chromatographic platform and timsTOF mass spectrometer. The DIA acquisition settings included positive ion detection and MS and MS/MS scan ranges of 100–1700m/z. MS^2^ spectra were acquired in DIA mode using four TIMS scan windows, each with an accumulation time of 100 ms. In PASEF mode, collision energy was varied linearly with ion mobility 1/K0, with fragmentation energy ranging from 20 to 59 eV across an ion mobility range of 0.85–1.30 Vs/cm^2^.

### 2.8 Protein alignment

DDA MS data were used to generate a DDA spectral library using Spectronaut software. Raw data were searched against the human UniProt reference proteome. Search parameters were as follows: enzyme, trypsin; maximum missed cleavage sites, 1; fixed modification, carbamidomethylation (C); and variable modifications, oxidation (M) and acetylation (protein N-terminus). Identified proteins were required to pass a false discovery rate (FDR) threshold of <1%. DIA data were also processed using Spectronaut software. Parameters included dynamic iRT-based retention time prediction, enabled MS2-level interference correction, enabled cross-run normalization, and protein and precursor FDR thresholds of 1%. Protein quantities were reported only for proteins passing the filter using the“Q-value sparse”mode.

### 2.9 Bioinformatics analysis

#### 2.9.1 Data filtering and normalization

The raw protein intensity matrix was matched to the sample annotation data. Samples with quantitative intensity values for fewer than 2,000 protein entries were excluded as low-coverage profiles using a study-specific operational cutoff based on the observed distribution of sample-level proteomic coverage. Proteins detected in at least 50% of samples in any predefined group were subsequently retained, followed by log2 transformation and median normalization. Remaining missing values were imputed separately for each sample using a left-shifted Gaussian distribution centered 1.8 sample-specific standard deviations below the observed mean, with a width of 0.3 standard deviations and a fixed random seed of 2026. As a sensitivity analysis, missing values were alternatively imputed using k-nearest-neighbor imputation (k = 10; random seed, 2026), after which NMF clustering and the principal differential-expression comparisons were repeated using the original settings and significance criteria.

#### 2.9.2 Differential expression analysis

Differential expression analysis was performed using the R package “limma” (V3.62.2). Unless otherwise stated, differentially expressed proteins were identified using the following criteria: adjusted *P* value < 0.05 and |log_2_FC| > log_2_(1.5).

#### 2.9.3 Unsupervised clustering

Non-negative matrix factorization (NMF), an unsupervised clustering algorithm (19, 20), was applied to identify proteomic subtypes within the Sepsis-only cohort using the R package “NMF” (V0.28). After matching proteomic profiles with clinical annotations and quality-control filtering, 96 Sepsis-only samples were included. The NMF input was derived from the globally preprocessed matrix without additional imputation. Proteins detected in at least 50% of samples were retained, and the 500 proteins with the highest median absolute deviation were selected and rescaled to the 0–1 range. Candidate ranks from k = 2 to k = 6 were evaluated using the Brunet algorithm with 100 runs per rank and a fixed random seed of 2026. Rank selection integrated consensus stability, reconstruction fit, consensus-matrix structure, cluster-size balance, and parsimony. Patient-resampling stability was assessed for k = 2 and k = 3 using 100 iterations of 80% subsampling with 10 NMF runs per iteration, and subtype memberships were compared across k = 2–6.

#### 2.9.4 Pathway enrichment analysis

GO and KEGG enrichment analyses were performed using the R package “clusterProfiler” (V4.14.6) and “org.Hs.eg.db” (V3.20.0). Significant enrichment was determined using adjusted *P* value < 0.05. Hallmark pathway activity was evaluated using GSVA with the R package “GSVA” (V2.0.7) based on MSigDB Hallmark gene sets. Differential GSVA scores between subtypes were compared using the Wilcoxon rank-sum test followed by Benjamini-Hochberg correction.

#### 2.9.5 Subtype projection analysis

To evaluate proteomic similarity between Sepsis-only-derived subtypes and sepsis-related organ dysfunction groups, a centroid-based projection analysis was performed. Subtype centroids were calculated as the median expression profile of each Sepsis-only subtype. Samples from S-AKI, S-ARDS, and S-ARDS&AKI groups were projected into the subtype space by calculating Pearson and Spearman correlations with each subtype centroid. Each sample was assigned to the closest subtype according to the highest correlation coefficient, following the general principle of nearest-centroid molecular subtype assignment (21, 22). This analysis was used as a *post hoc* similarity analysis and was not used to define the original NMF subtypes. Pearson and Spearman correlations with both subtype centroids were calculated, and an absolute difference between the paired centroid correlations of <0.05 was operationally considered an ambiguous projection.

#### 2.9.6 Clinical adjustment and sensitivity analyses

Multivariable linear regression with HC3 robust standard errors was used to compare standardized S3-like similarity margins between organ dysfunction phenotypes and the corresponding S1 or S2 reference group. The primary model adjusted for APACHE II score, septic shock, and chronic-comorbidity burden. Mechanical ventilation and CRRT were added in an organ-support sensitivity model. Additional analyses used SOFA instead of APACHE II, vasopressor use instead of septic shock, Spearman-based margins, exclusion of the S-ARDS&AKI group, and the entire Sepsis-only cohort as the reference. Results are reported as adjusted beta coefficients with 95% confidence intervals. *P* values were corrected using the Benjamini–Hochberg method within each prespecified model family.

#### 2.9.7 Machine learning-based construction of a protein panel

Elastic net logistic regression was used to construct a protein panel from EV-enriched proteomic features using the R package “glmnet” (v4.1–10). Candidate proteins were defined as shared up-regulated EV-associated proteins in the S3 vs S2 and Sepsis&Complications vs S2 comparisons. Sepsis&Complications comprised the S-AKI, S-ARDS, and S-ARDS&AKI groups. The model was developed to distinguish S2 from Sepsis&Complications. Alpha values from 0.1 to 0.9 were evaluated, and lambda was selected using the lambda.1se rule based on cross-validated binomial deviance. Proteins with non-zero coefficients in the full-cohort model were retained in the protein panel and used to calculate predicted probabilities from the full-cohort elastic net model for all samples. Supportive repeated cross-validation and fixed-panel modelling were performed using “caret” (v7.0–10), with AUROC and related classification metrics calculated using “pROC” (v1.19.0.1).

#### 2.9.8 Repeated nested internal validation of the downstream supervised workflow

The previously derived NMF subtype labels were treated as fixed exploratory phenotype labels. The downstream supervised candidate-selection and elastic-net modelling workflow was evaluated using 100 repetitions of stratified five-fold nested cross-validation. Outer partitions were stratified by analysis group. Within each outer training partition, protein detection filtering, missing-value imputation, differential expression analyses for S3 vs S2 and Sepsis&Complications vs S2, shared-candidate selection, predictor scaling, model tuning, and decision-threshold derivation were repeated without access to the corresponding outer test samples. S3, S2, and Sepsis&Complications samples assigned to an outer test fold were not used for candidate selection or model fitting in that fold. Elastic-net parameters were selected by stratified inner cross-validation, and decision thresholds were derived from inner out-of-fold probabilities using the Youden index. Sepsis&Complications vs S2 was the primary task, and S3 vs S2 was the secondary task.

Discrimination, calibration, classification performance, decision-threshold stability, and protein-selection frequency were evaluated using outer out-of-fold predictions. Within each outer fold, calibration parameters were estimated from training-derived out-of-fold predictions and applied to the corresponding outer-test probabilities. Decision-curve analysis was performed using the patient-aggregated recalibrated out-of-fold probabilities, with treat-all and treat-none strategies as references. Performance estimates were averaged across the 100 repetitions. Confidence intervals were obtained from 2,000 stratified patient-level bootstrap resamples. AUROC and PR-AUC were additionally corrected for optimism using 1,000 bootstrap resamples of the downstream supervised workflow.

### 2.10 Statistical analysis

All statistical analyses were conducted using R (v4.4.3). Continuous variables were compared using the Kruskal–Wallis test, while categorical variables were analyzed using the chi-square test or Fisher's exact test, as appropriate. *Post hoc* pairwise subtype comparisons were performed using the Wilcoxon rank-sum test or Fisher's exact test, with *P* values adjusted using the Benjamini–Hochberg method. Overall clinical comparisons were considered descriptive and were not adjusted for multiple comparisons. All tests were two-sided, and statistical significance was defined as *P* or adjusted *P* < 0.05.

## 3 Results

### 3.1 Study cohort and characterization of serum EV-enriched preparations

The study design and analytical workflow are summarized in Figure 1. A total of 250 patients with sepsis and 50 healthy controls were initially enrolled. Twenty-one samples with fewer than 2,000 identified proteins were excluded according to the sample-level quality-control criterion, including 2 healthy controls, 4 Sepsis-only patients, 4 S-ARDS patients, 2 S-AKI patients, and 9 S-ARDS&AKI patients. The final analysis included 279 samples: 48 healthy controls, 96 Sepsis-only patients, 46 S-ARDS patients, 48 S-AKI patients, and 41 S-ARDS&AKI patients. The clinical characteristics of the 231 patients with sepsis are summarized in Table 1. The median age was 69.00 years [IQR, 58.00–77.00], and 80 patients were female (34.6%). The median APACHE II and SOFA scores were 19.00 [15.00–23.00] and 6.00 [4.00–9.00], respectively. Septic shock occurred in 75 patients (32.5%), and the overall 28-day mortality rate was 28.6% (66/231).

**Figure 1:**
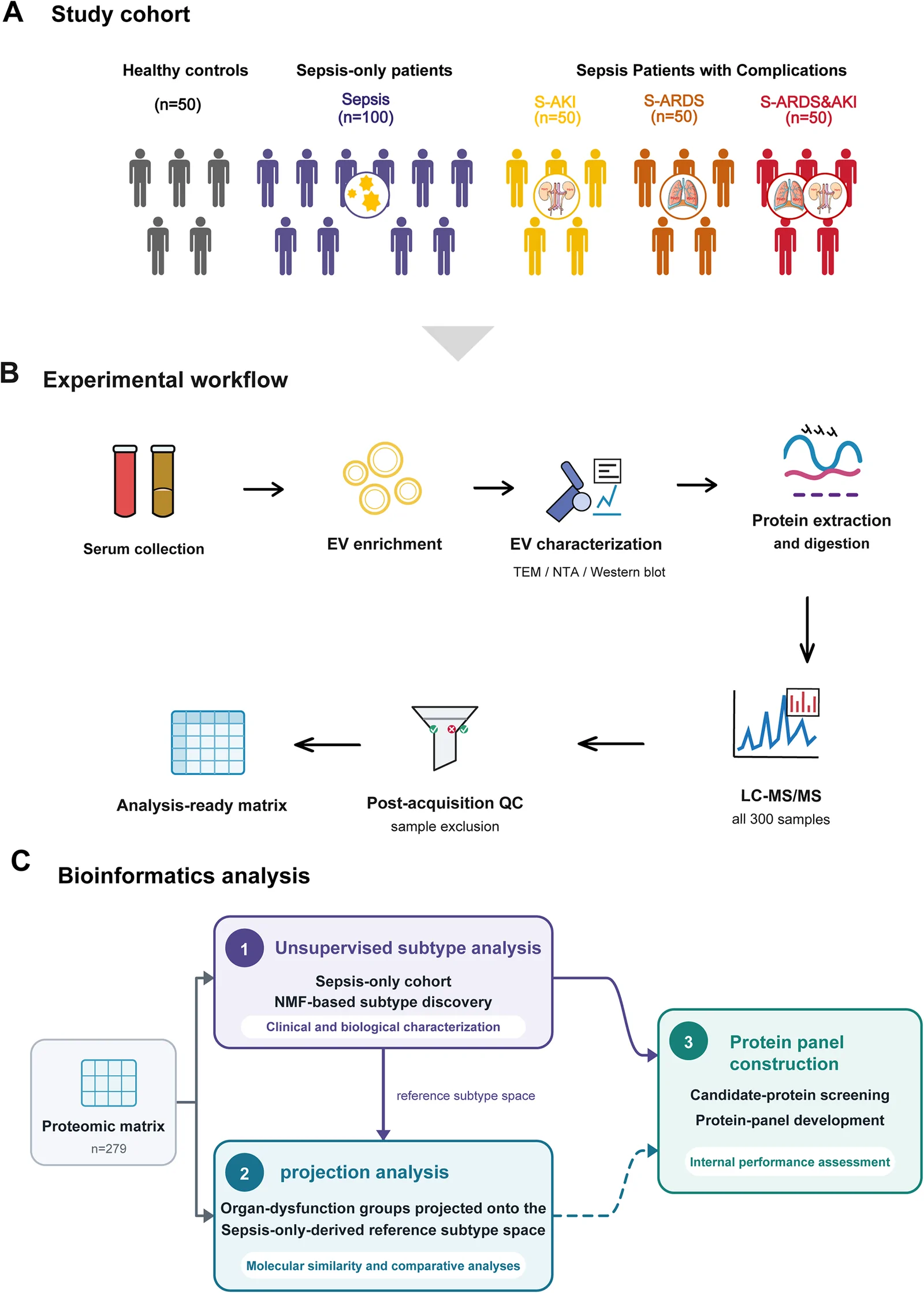
Study workflow. **(A)** Patients enrolled in each group. **(B)** Workflow of serum EV-enriched proteomic analysis. **(C)** Bioinformatics analysis comprising NMF-based unsupervised clustering, differential expression and pathway enrichment analyses, subtype projection, clinical adjustment and sensitivity analyses, protein panel construction, and repeated nested internal validation.

**Table 1:** Clinical characteristics and outcomes of sepsis patients included in the final proteomic analysis.

Variable	Overall (*n* = 231)	Sepsis-only (*n* = 96)	S-ARDS (*n* = 46)	S-AKI (*n* = 48)	S-ARDS&AKI (*n* = 41)	*P value*
Demographics						
Age, years	69.00 [58.00, 77.00]	69.00 [57.75, 77.00]	69.50 [52.00, 76.75]	68.00 [61.00, 75.50]	70.00 [59.00, 76.00]	0.925
Female, n/N (%)	80/231 (34.6%)	29/96 (30.2%)	15/46 (32.6%)	17/48 (35.4%)	19/41 (46.3%)	0.333
BMI, kg/m²	23.40 [21.60, 24.95]	23.30 [21.55, 24.57]	23.40 [21.52, 25.32]	22.68 [21.28, 24.80]	23.89 [22.80, 25.56]	0.467
Severity of disease on admission						
APACHE II score	19.00 [15.00, 23.00]	17.00 [13.75, 20.25]	19.00 [16.00, 21.00]	20.50 [16.00, 27.25]	19.00 [17.00, 25.00]	<0.001
SOFA score	6.00 [4.00, 9.00]	5.00 [4.00, 7.00]	6.00 [4.00, 7.00]	8.00 [5.75, 10.25]	11.00 [7.00, 12.00]	<0.001
Septic shock, n/N (%)	75/231 (32.5%)	18/96 (18.8%)	14/46 (30.4%)	19/48 (39.6%)	24/41 (58.5%)	<0.001
Chronic comorbidities						
Cardiovascular disease, n/N (%)	70/231 (30.3%)	22/96 (22.9%)	12/46 (26.1%)	23/48 (47.9%)	13/41 (31.7%)	0.019
Chronic pulmonary disease, n/N (%)	29/231 (12.6%)	8/96 (8.3%)	5/46 (10.9%)	10/48 (20.8%)	6/41 (14.6%)	0.184
Diabetes mellitus, n/N (%)	56/231 (24.2%)	21/96 (21.9%)	8/46 (17.4%)	18/48 (37.5%)	9/41 (22.0%)	0.103
Cerebrovascular disease, n/N (%)	60/231 (26.0%)	28/96 (29.2%)	5/46 (10.9%)	17/48 (35.4%)	10/41 (24.4%)	0.041
Immune system disease, n/N (%)	7/231 (3.0%)	2/96 (2.1%)	2/46 (4.3%)	2/48 (4.2%)	1/41 (2.4%)	0.818
Chronic liver disease, n/N (%)	5/231 (2.2%)	0/96 (0.0%)	1/46 (2.2%)	4/48 (8.3%)	0/41 (0.0%)	0.009
Chronic kidney disease, n/N (%)	6/231 (2.6%)	2/96 (2.1%)	0/46 (0.0%)	3/48 (6.2%)	1/41 (2.4%)	0.299
Solid tumor, n/N (%)	22/231 (9.5%)	14/96 (14.6%)	0/46 (0.0%)	4/48 (8.3%)	4/41 (9.8%)	0.025
Hematologic disorder, n/N (%)	2/231 (0.9%)	0/96 (0.0%)	0/46 (0.0%)	2/48 (4.2%)	0/41 (0.0%)	0.112
First-day organ support and treatment						
Mechanical ventilation, n/N (%)	138/225 (61.3%)	46/94 (48.9%)	36/42 (85.7%)	18/48 (37.5%)	38/41 (92.7%)	<0.001
Continuous renal replacement therapy, n/N (%)	66/231 (28.6%)	8/96 (8.3%)	8/46 (17.4%)	18/48 (37.5%)	32/41 (78.0%)	<0.001
Vasopressors, n/N (%)	104/231 (45.0%)	25/96 (26.0%)	19/46 (41.3%)	27/48 (56.2%)	33/41 (80.5%)	<0.001
Laboratory findings at sampling						
White blood cell count, × 10^9/L	10.26 [6.08, 15.12]	10.30 [6.84, 14.81]	9.83 [7.02, 13.34]	11.29 [5.15, 18.43]	9.91 [4.96, 16.18]	0.823
Neutrophil count, × 10^9/L	9.11 [5.10, 13.52]	9.11 [5.12, 13.48]	8.77 [5.99, 11.54]	10.40 [4.66, 17.47]	8.77 [4.41, 12.97]	0.600
Neutrophil ratio, %	87.20 [82.10, 92.50]	86.85 [82.10, 92.53]	86.85 [83.17, 90.07]	91.15 [85.05, 93.40]	85.50 [79.20, 90.70]	0.027
Lymphocyte count, × 10^9/L	0.61 [0.38, 1.04]	0.63 [0.37, 0.96]	0.78 [0.49, 1.15]	0.51 [0.36, 0.79]	0.57 [0.35, 1.13]	0.220
Lymphocyte ratio, %	7.10 [4.30, 11.20]	6.90 [4.27, 11.22]	8.00 [5.40, 10.45]	4.98 [3.65, 10.90]	8.20 [5.10, 13.40]	0.186
Platelet count, × 10^9/L	138.00 [80.50, 196.50]	150.50 [100.00, 218.50]	153.50 [90.50, 204.00]	128.50 [66.25, 166.75]	82.00 [39.00, 143.00]	<0.001
Hemoglobin, g/L	96.00 [83.00, 114.50]	103.00 [85.75, 121.25]	93.00 [83.50, 112.75]	97.50 [79.75, 115.75]	88.00 [79.00, 99.00]	0.023
Procalcitonin, ng/mL	4.09 [1.02, 15.90]	2.64 [0.78, 7.70]	1.25 [0.27, 2.81]	17.96 [5.85, 51.00]	15.48 [4.26, 39.12]	<0.001
PaO₂/FiO₂, mmHg	226.50 [162.25, 319.50]	262.00 [201.00, 342.35]	157.50 [104.00, 203.75]	322.50 [231.75, 376.00]	182.00 [131.00, 247.00]	<0.001
Creatinine, μmol/L	88.00 [61.40, 139.40]	72.00 [54.08, 90.00]	61.70 [47.80, 80.30]	158.00 [121.75, 226.62]	154.60 [123.30, 192.60]	<0.001
Lactate, mmol/L	1.80 [1.20, 2.70]	1.60 [1.15, 2.40]	1.50 [1.20, 2.25]	2.40 [1.55, 4.75]	2.70 [1.95, 6.55]	<0.001
NT-proBNP, pg/mL	2,156.00 [552.75, 6,525.00]	1,395.00 [328.93, 4,210.25]	1,190.50 [363.25, 2,688.75]	4,950.00 [1,780.00, 8,940.00]	5,697.00 [1,270.00, 13,600.00]	<0.001
Outcomes						
ICU length of stay, days	8.00 [4.00, 12.00]	7.00 [3.00, 11.00]	9.00 [5.00, 13.00]	7.00 [4.00, 10.00]	10.00 [6.00, 16.00]	0.083
28-day mortality, n/N (%)	66/231 (28.6%)	22/96 (22.9%)	10/46 (21.7%)	14/48 (29.2%)	20/41 (48.8%)	0.013

Data are presented as median [IQR] or n/N (%). First-day variables were recorded during the first 24 h after ICU admission. Percentages were calculated using non-missing values as denominators. Mechanical ventilation data were missing for 6 patients; therefore, the denominator for this variable was 225 rather than 231. Continuous variables were compared using the Kruskal–Wallis test, and categorical variables were compared using the chi-square test or Fisher's exact test, as appropriate. *P* values are descriptive and were not adjusted for multiple comparisons. Variables closely related to the definitions or clinical management of ARDS and AKI, including PaO₂/FiO₂, creatinine, mechanical ventilation, and continuous renal replacement therapy, should be interpreted descriptively. No imputation was performed for baseline clinical variables.

The enrichment procedure is illustrated in Figure 2A. Immunoblotting detected the EV-associated markers CD9, CD81, and TSG101 in representative serum EV-enriched preparations, whereas Calnexin showed no detectable signal and was readily detected in the cell-lysate positive control (23, 24) (Figure 2B). TEM showed cup-shaped or concave EV-like vesicular structures, and NTA indicated that the detected particles were predominantly 60–100 nm in diameter (Figures 2C,D). Of the 4,754 quantified protein groups, 4,747 had gene-symbol annotations and were used for comparison with the ExoCarta Top 100 EV proteins; 95 of these reference proteins were identified in the present dataset (25) (Figure 2E). Proteins included in two published EV-associated marker panels (26) were also detected across all five clinical groups (Figure 2F). These findings were consistent with enrichment of EV-associated material from serum. In the proteomic data, CD81 was more abundant in each sepsis group than in healthy controls, whereas CD9 and CD63 showed lower abundance (Figure 2G). PDCD6IP (ALIX) and TSG101 were also more abundant in the sepsis groups (Figure 2H). HSPA4, HSPA8, HSP90AA1, and HSP90AB1 showed higher abundance in the S-ARDS&AKI group, while FLOT1 and FLOT2 were more abundant in the sepsis groups than in controls (Figures 2I,J).

**Figure 2:**
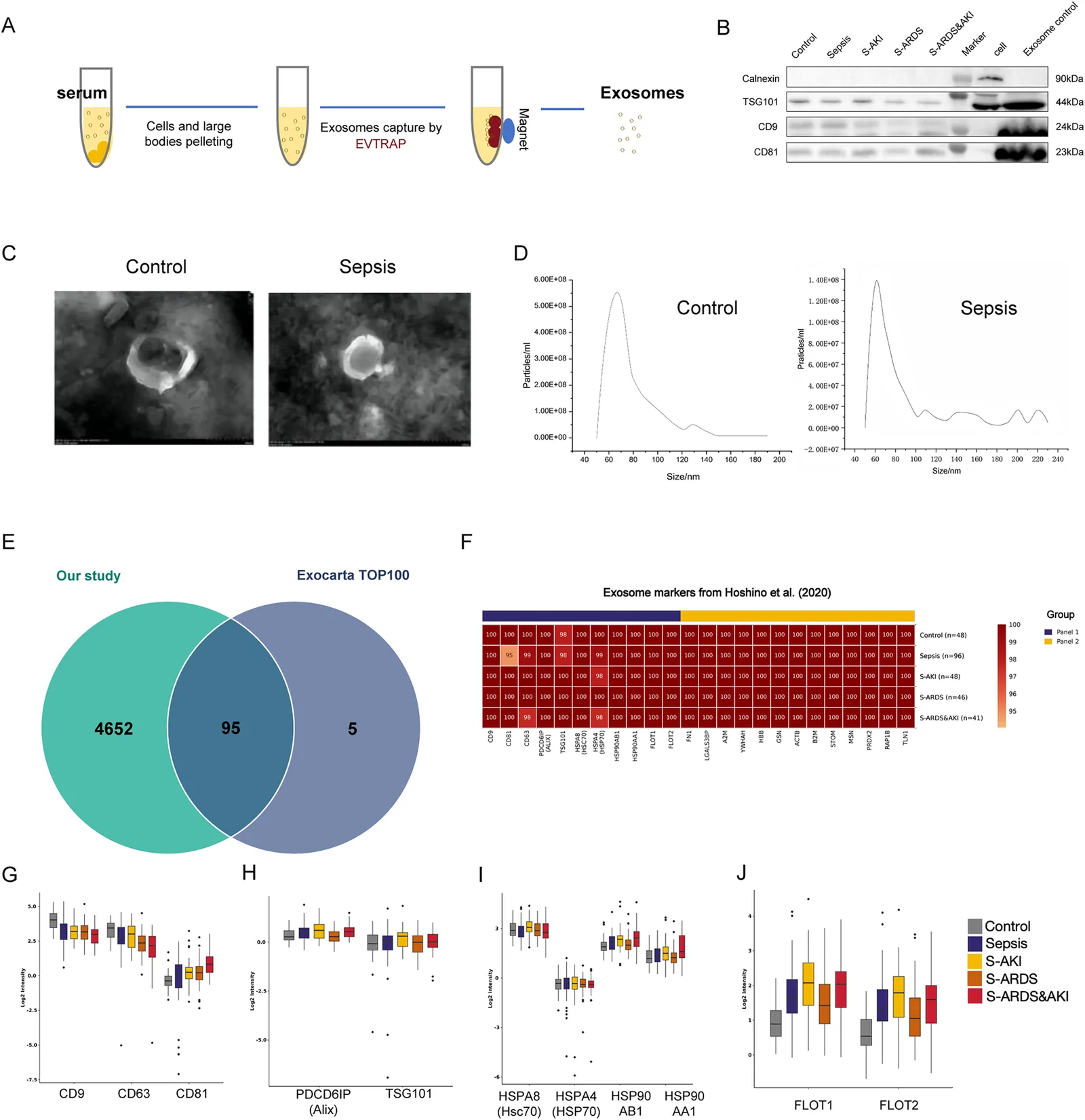
Characterization of serum EV-enriched preparations. **(A)** Workflow of EV enrichment. **(B)** Western blot analysis of CD81, CD9, Calnexin and TSG101. **(C,D)** TEM images **(C)** and NTA size distribution **(D)** of EV-like vesicular structures derived from serum samples. **(E)** Gene-symbol-based overlap between the 4,747 annotated protein groups identified in this study and the ExoCarta Top 100 EV proteins. **(F)** EV-associated proteins from two established marker panels identified in this study. **(G–J)** Relative abundance of representative EV-associated and inflammation-related proteins across groups, including tetraspanins **(G)**, PDCD6IP/TSG101 **(H)**, inflammation-related heat shock proteins **(I)**, and FLOT1/FLOT2 **(J)**.

### 3.2 Serum EV-enriched proteomic differences across clinical groups

Following quality-control filtering, 4,754 protein groups were quantified across 279 samples. On average, 2,796 protein groups were identified per sample, ranging from 2,005 in the S-ARDS&AKI group to 3,840 in the Sepsis-only group (Figure 3A). Principal component analysis (PCA) of the EV-enriched proteomic data separated healthy controls from sepsis-related samples but did not clearly distinguish Sepsis-only, S-AKI, S-ARDS, and S-ARDS&AKI subgroups from one another (Figure 3B). This pattern highlights the molecular overlap and heterogeneity of sepsis-related clinical phenotypes. Differential EV-associated protein analysis was then performed between controls and Sepsis-only samples. Compared with controls, 421 proteins were significantly up-regulated and 551 proteins were significantly down-regulated in Sepsis-only samples (Figure 3C). KEGG pathway enrichment analysis of the up-regulated proteins identified several pathways, including ECM–receptor interaction, integrin signaling, PI3K–Akt signaling, HIF-1 signaling, focal adhesion, phagosome, and complement and coagulation cascades, among others (Figure 3D). Representative pathways enriched among the down-regulated proteins included IgSF CAM signaling, platelet activation, regulation of the actin cytoskeleton, tight junction, focal adhesion, Fc gamma R-mediated phagosome formation, and several infection-related pathways (Figure 3E).

**Figure 3:**
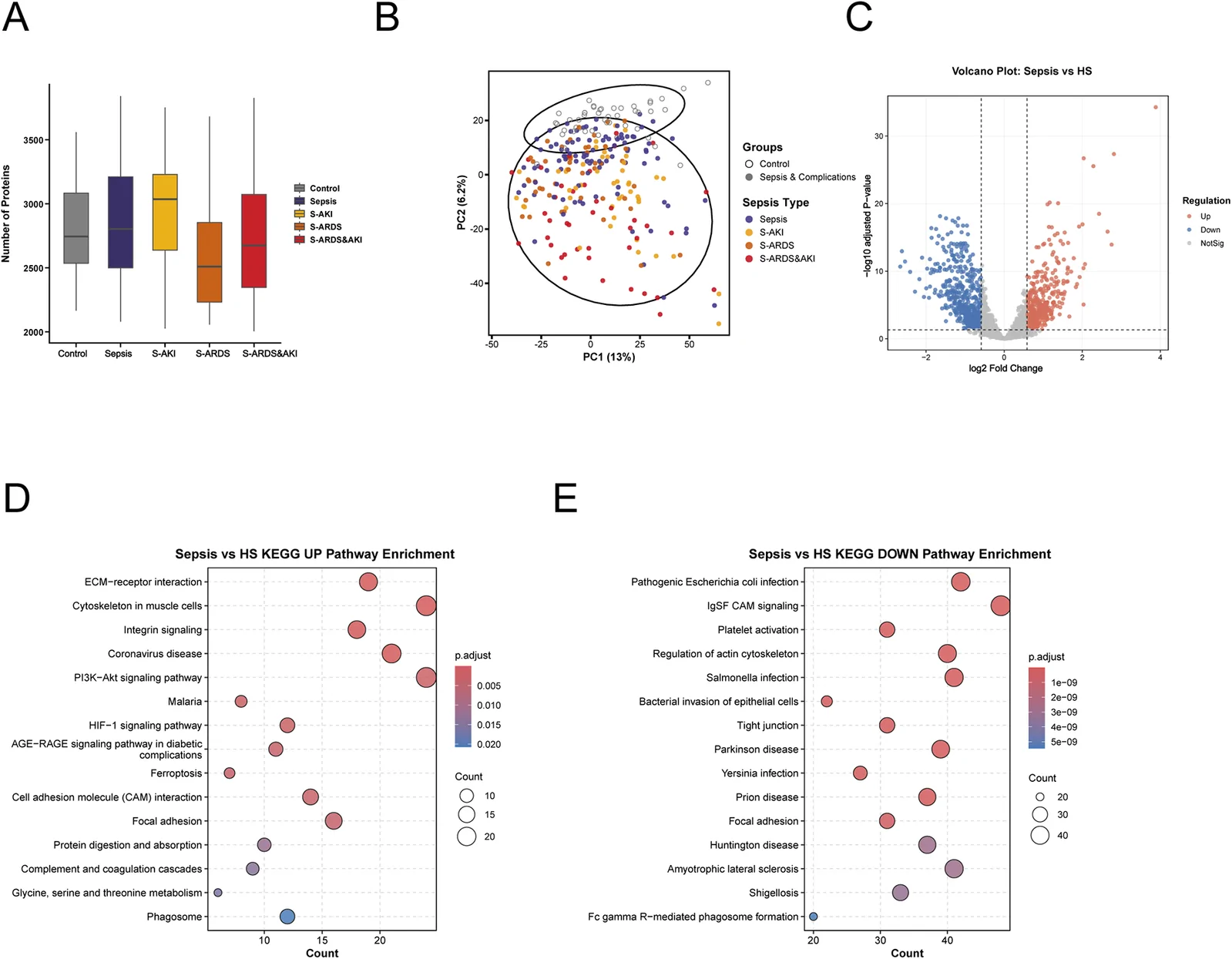
Serum EV-enriched proteomic analysis of sepsis and sepsis-associated complications. **(A)** Number of identified proteins across the five groups. **(B)** Principal component analysis (PCA) of serum EV-enriched proteomic profiles. **(C)** Volcano plot showing differentially expressed EV-associated proteins between controls and Sepsis-only samples. **(D,E)** KEGG pathway enrichment analyses of up-regulated **(D)** and down-regulated **(E)** proteins.

### 3.3 NMF identifies three serum EV-enriched proteomic subtypes within the sepsis-only cohort

To investigate proteomic heterogeneity within the Sepsis-only cohort, NMF consensus clustering was applied to serum EV-enriched proteomic profiles from 96 patients. Although k = 2 showed the strongest consensus stability, k = 3 retained high cophenetic correlation and consensus silhouette values, showed the largest relative increase in CDF area, and reduced the RSS relative to k = 2 (Figure 4A; Supplementary Table S1). Patient-resampling stability was lower for k = 3 than for k = 2 (median ARI, 0.614 vs. 0.751). We therefore retained k = 3 as an exploratory higher-resolution solution, yielding S1 (*n* = 26), S2 (*n* = 33), and S3 (*n* = 37) (Figure 4B). All 37 S3 patients were contained within the k = 2 C2 cluster, which retained the humoral immune and complement/coagulation signature, whereas S3 membership was progressively partitioned at higher ranks (Supplementary Figure S1; Supplementary Table S1).

**Figure 4:**
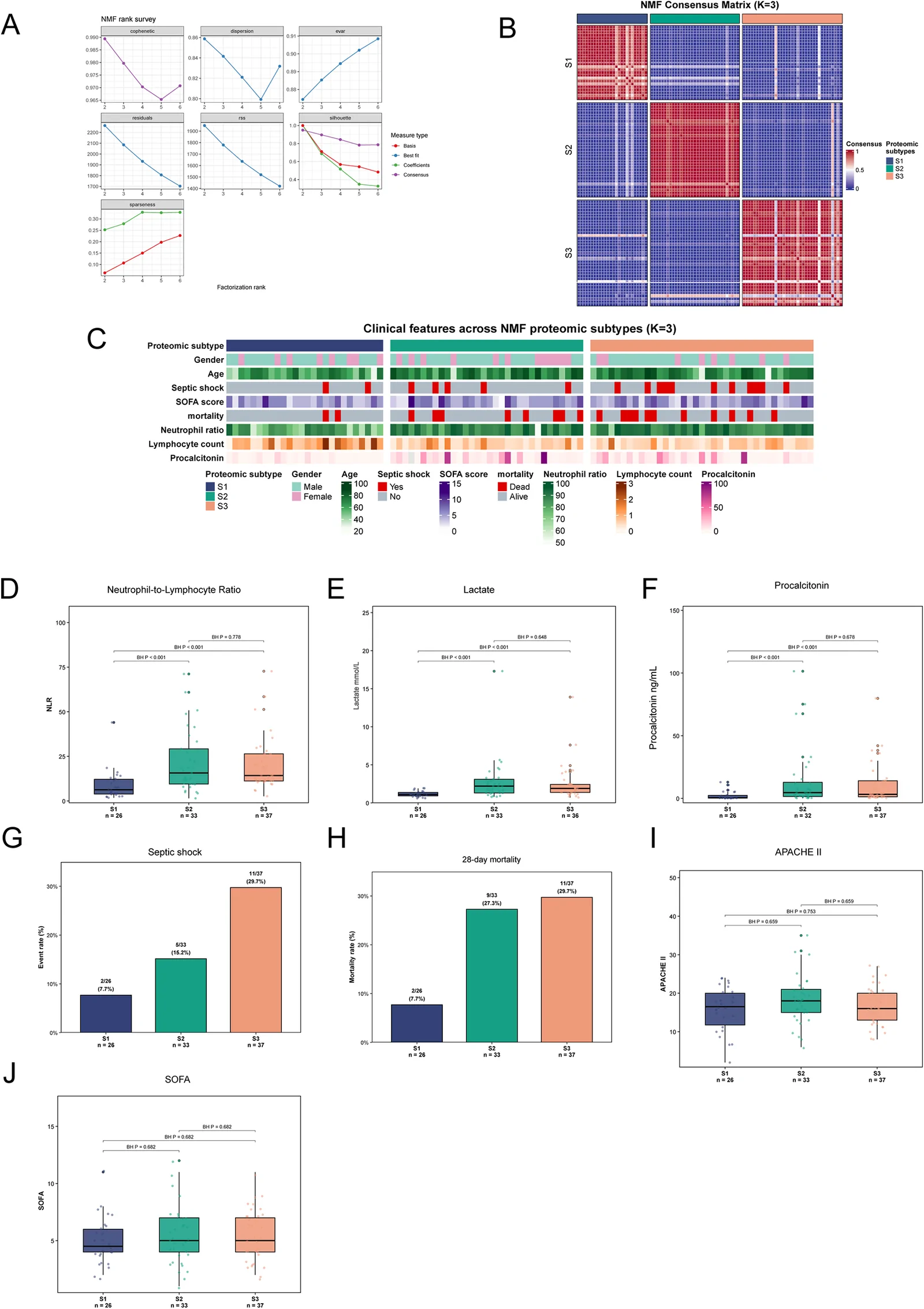
Molecular subtyping of sepsis-only patients based on serum EV-enriched proteomics. **(A)** NMF rank-survey metrics used for multi-criterion evaluation of candidate factorization ranks. **(B)** Consensus matrix for the exploratory three-subtype solution at k = 3. **(C)** Clinical feature heatmap across S1, S2, and S3. **(D–J)** Comparisons of clinical indicators among the three subtypes, including neutrophil-to-lymphocyte ratio, lactate, procalcitonin, septic shock, 28-day mortality, APACHE II score, and SOFA score.

Compared with S1, S2 and S3 showed higher neutrophil-to-lymphocyte ratios, lactate concentrations, and procalcitonin levels, whereas APACHE II and SOFA scores overlapped substantially across the subtypes (Figures 4C–J; Table 2). Septic shock and 28-day mortality were descriptively more frequent in S2 and S3. Acquisition batch was not significantly associated with subtype membership (Fisher's exact *P* = 0.547; Cramér's V = 0.126). The batch-adjusted NMF solution showed 79.2% agreement with the original assignments (ARI, 0.520), with greater retention of S2 and S3 than S1. Together with the technical QC and PERMANOVA results, these findings indicate that acquisition batch was not the sole driver of the central S2/S3 structure, although some assignment sensitivity remained (Supplementary Figure S2; Supplementary Table S2).

**Table 2:** Clinical characteristics and outcomes of the three EV-enriched proteomic subtypes in sepsis-only patients.

Variable	Overall (*n* = 96)	S1 (*n* = 26)	S2 (*n* = 33)	S3 (*n* = 37)	*P-*value
Demographics					
Age, years, median [IQR]	69.00 [57.75, 77.00]	61.00 [50.00, 73.00]	73.00 [63.00, 79.00]	71.00 [63.00, 78.00]	0.015
Female, n/N (%)	29/96 (30.2%)	8/26 (30.8%)	13/33 (39.4%)	8/37 (21.6%)	0.270
BMI, kg/m2, median [IQR]	23.30 [21.55, 24.57]	23.37 [22.20, 24.73]	23.70 [22.92, 24.44]	22.92 [20.66, 24.20]	0.250
Severity of disease on admission					
APACHE II score, median [IQR]	17.00 [13.75, 20.25]	16.50 [11.75, 20.00]	18.00 [15.00, 21.00]	16.00 [13.00, 20.00]	0.627
SOFA score, median [IQR]	5.00 [4.00, 7.00]	4.50 [4.00, 6.00]	5.00 [4.00, 7.00]	5.00 [4.00, 7.00]	0.698
Septic shock, n/N (%)	18/96 (18.8%)	2/26 (7.7%)	5/33 (15.2%)	11/37 (29.7%)	0.079
Chronic comorbidities					
Cardiovascular disease, n/N (%)	22/96 (22.9%)	8/26 (30.8%)	4/33 (12.1%)	10/37 (27.0%)	0.179
Chronic pulmonary disease, n/N (%)	8/96 (8.3%)	1/26 (3.8%)	1/33 (3.0%)	6/37 (16.2%)	0.124
Diabetes mellitus, n/N (%)	21/96 (21.9%)	5/26 (19.2%)	7/33 (21.2%)	9/37 (24.3%)	0.885
Cerebrovascular disease, n/N (%)	28/96 (29.2%)	6/26 (23.1%)	10/33 (30.3%)	12/37 (32.4%)	0.712
Immune system disease, n/N (%)	2/96 (2.1%)	2/26 (7.7%)	0/33 (0.0%)	0/37 (0.0%)	0.070
Chronic liver disease, n/N (%)	0/96 (0.0%)	0/26 (0.0%)	0/33 (0.0%)	0/37 (0.0%)	-
Chronic kidney disease, n/N (%)	2/96 (2.1%)	1/26 (3.8%)	1/33 (3.0%)	0/37 (0.0%)	0.521
Solid tumor, n/N (%)	14/96 (14.6%)	1/26 (3.8%)	9/33 (27.3%)	4/37 (10.8%)	0.034
Hematologic disorder, n/N (%)	0/96 (0.0%)	0/26 (0.0%)	0/33 (0.0%)	0/37 (0.0%)	-
First-day organ support and treatment					
Mechanical ventilation, n/N (%)	46/94 (48.9%)	14/26 (53.8%)	11/32 (34.4%)	21/36 (58.3%)	0.120
Continuous renal replacement therapy, n/N (%)	8/96 (8.3%)	3/26 (11.5%)	2/33 (6.1%)	3/37 (8.1%)	0.811
Vasopressors, n/N (%)	25/96 (26.0%)	4/26 (15.4%)	8/33 (24.2%)	13/37（35.1%）	0.204
Laboratory findings at sampling					
White blood cell count, ×10^9/L	10.30 [6.84, 14.81]	7.62 [4.74, 13.97]	12.11 [8.00, 15.04]	10.60 [7.53, 13.20]	0.221
Neutrophil count, ×10^9/L	9.11 [5.12, 13.48]	5.86 [3.84, 11.56]	10.67 [6.86, 14.41]	9.31 [5.69, 12.06]	0.100
Neutrophil ratio, %	86.85 [82.10, 92.53]	82.20 [70.58, 86.07]	87.80 [83.10, 94.10]	90.10 [85.30, 92.80]	<0.001
Lymphocyte count, ×10^9/L	0.63 [0.37, 0.96]	1.03 [0.63, 1.47]	0.60 [0.34, 0.74]	0.52 [0.34, 0.70]	0.004
Lymphocyte ratio, %	6.90 [4.27, 11.22]	13.10 [7.15, 18.53]	6.70 [3.20, 9.40]	6.20 [3.50, 7.70]	<0.001
Platelet count, ×10^9/L	150.50 [100.00, 218.50]	163.50 [100.75, 241.00]	143.00 [92.00, 234.00]	145.00 [100.00, 196.00]	0.791
Hemoglobin, g/L	103.00 [85.75, 121.25]	98.50 [80.00, 110.25]	106.00 [91.00, 125.00]	103.00 [86.00, 120.00]	0.394
C-reactive protein, mg/L	110.10 [66.55, 161.08]	131.70 [74.44, 152.70]	107.00 [62.25, 152.95]	111.95 [70.23, 177.35]	0.756
Procalcitonin, ng/mL	2.64 [0.78, 7.70]	0.49 [0.18, 2.34]	4.60 [1.46, 12.83]	3.23 [1.20, 14.00]	<0.001
PaO2/FiO2 ratio, mmHg	262.00 [201.00, 342.35]	290.00 [202.93, 363.25]	275.00 [192.00, 360.00]	247.00 [203.00, 306.75]	0.483
Creatinine, μmol/L	72.00 [54.08, 90.00]	60.30 [47.72, 79.67]	78.50 [58.50, 89.00]	70.00 [59.70, 104.40]	0.140
Lactate, mmol/L	1.60 [1.15, 2.40]	1.10 [0.92, 1.37]	2.20 [1.30, 3.10]	1.90 [1.37, 2.42]	<0.001
NT-proBNP, pg/mL	1,395.00 [328.93, 4,210.25]	771.75 [164.75, 2,315.00]	1,480.00 [538.35, 5,144.50]	2,210.00 [599.00, 5,350.00]	0.080
Outcomes					
ICU length of stay, days	7.00 [3.00, 11.00]	10.00 [5.00, 14.50]	6.00 [2.10, 9.00]	7.00 [4.00, 11.00]	0.024
28-day mortality, n/N (%)	22/96 (22.9%)	2/26 (7.7%)	9/33 (27.3%)	11/37 (29.7%)	0.094

Data are presented as median [IQR] or n/N (%). First-day variables were recorded during the first 24 h after ICU admission. Percentages were calculated using non-missing observations as denominators. Mechanical ventilation status was missing for two patients, including one patient in S2 and one patient in S3; vasopressor-use data were missing for two patients in S3. Continuous variables were compared using the Kruskal–Wallis test, and categorical variables were compared using the chi-square test or Fisher's exact test, as appropriate. *P* values are descriptive and were not adjusted for multiple comparisons. *P* values were not calculated for variables with no events in any group. No imputation was performed for clinical variables.

### 3.4 Differential protein and pathway analyses distinguish the S2 and S3 proteomic profiles

Differential protein analysis was performed to characterize the molecular differences between S2 and S3. A total of 1,440 proteins differed significantly between the two subtypes, including 116 proteins with higher abundance and 1,324 proteins with lower abundance in S3 relative to S2 (Figure 5A).

**Figure 5:**
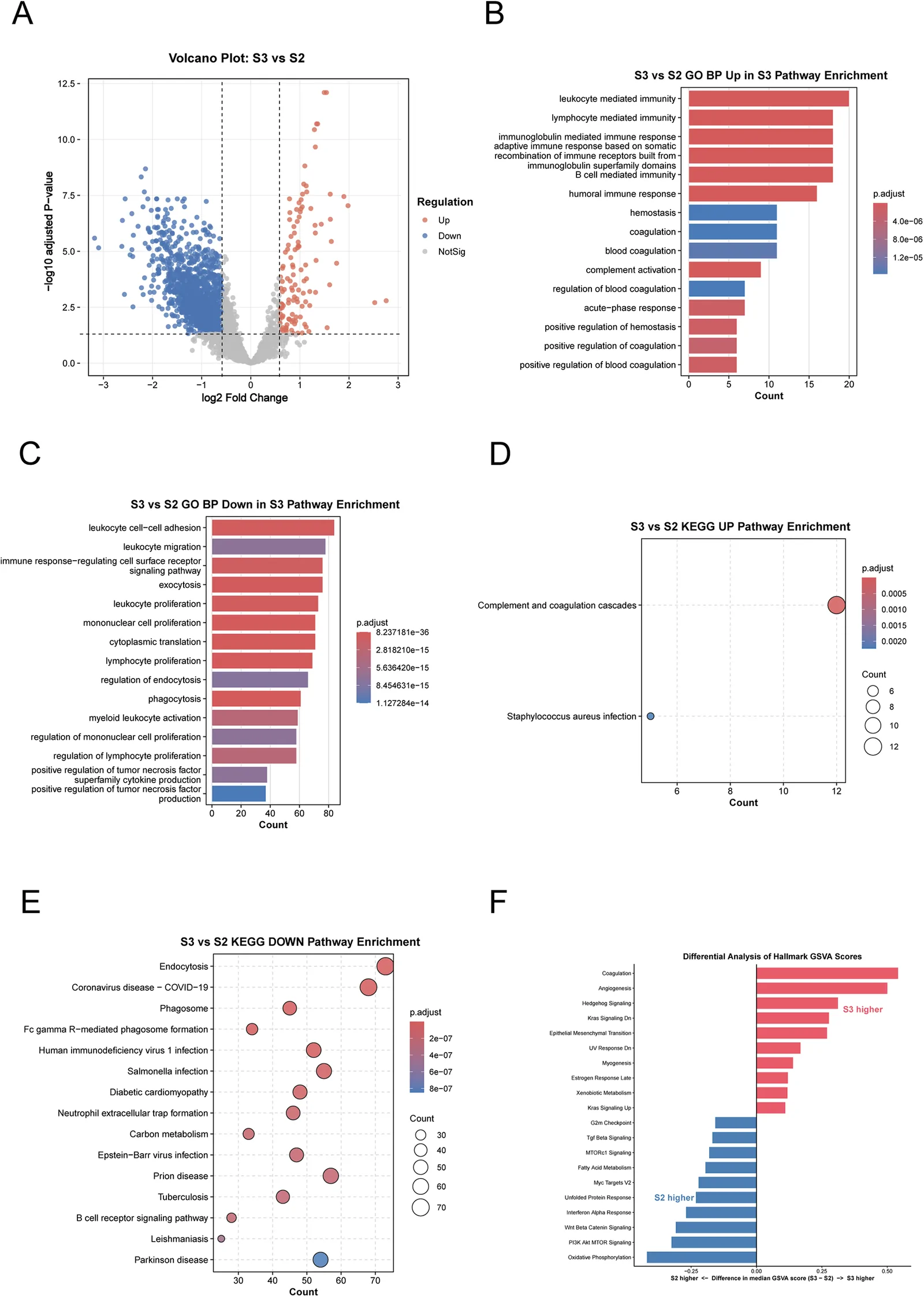
Differential EV-associated proteins, pathway enrichment, and hallmark GSVA scores distinguishing S3 from S2. **(A)** Volcano plot showing differentially expressed EV-associated proteins between S3 and S2. Proteins with higher levels in S3 are shown in red, proteins with lower levels in S3 are shown in blue, and non-significant proteins are shown in gray. **(B)** GO Biological Process enrichment analysis of proteins up-regulated in S3 compared with S2. **(C)** GO Biological Process enrichment analysis of proteins down-regulated in S3 compared with S2. **(D)** KEGG pathway enrichment analysis of proteins up-regulated in S3 compared with S2. **(E)** KEGG pathway enrichment analysis of proteins down-regulated in S3 compared with S2. **(F)** Differential analysis of Hallmark GSVA scores between S3 and S2. Positive values indicate higher median GSVA scores in S3, whereas negative values indicate higher median GSVA scores in S2.

Representative GO biological processes enriched among proteins with higher abundance in S3 included humoral, immunoglobulin-mediated, and B-cell-mediated immunity, complement activation, coagulation, hemostasis, and the acute-phase response (Figure 5B). Representative KEGG pathways enriched among proteins with higher abundance in S3 included complement and coagulation cascades and the *Staphylococcus aureus* infection pathway (Figure 5D). Proteins with lower abundance in S3 were enriched in representative processes related to leukocyte adhesion and migration, immune receptor signaling, exocytosis, phagocytosis, and myeloid leukocyte activation (Figure 5C). Representative KEGG pathways included endocytosis, phagosome, Fc gamma R-mediated phagosome formation, neutrophil extracellular trap formation, and B-cell receptor signaling, among others (Figure 5E). Among the Hallmark gene sets displayed, coagulation showed a higher median GSVA score in S3, whereas oxidative phosphorylation, PI3K–AKT–mTOR signaling, interferon-α response, unfolded protein response, and mTORC1 signaling were representative gene sets with higher scores in S2 (Figure 5F).

The S3 vs S1 comparison showed a similar contrast: proteins with higher abundance in S3 were enriched in representative humoral immune, B-cell, complement, and acute-phase processes, whereas proteins with lower abundance were primarily enriched in translation- and ribosome-related processes (Supplementary Figure S3).

### 3.5 Organ dysfunction groups show partial similarity to the S3 proteomic profile

To examine whether the proteomic patterns identified in the Sepsis-only cohort were also present in patients with established organ dysfunction, samples from the S-AKI, S-ARDS, and S-ARDS&AKI groups were projected into the S3 vs S1 and S3 vs S2 centroid spaces. In both projection spaces, a proportion of patients in each organ dysfunction group showed a higher correlation with the S3 centroid than with the corresponding S1 or S2 centroid (Figures 6A,B). Sample-level Pearson and Spearman correlation distributions and ambiguous projection rates are reported in Supplementary Figure S4 and Supplementary Table S3. Overlap was substantially greater between the S2 and S3 centroids than between S1 and S3, indicating that the S3 vs S2 projection provided limited separation at the individual-patient level.

**Figure 6:**
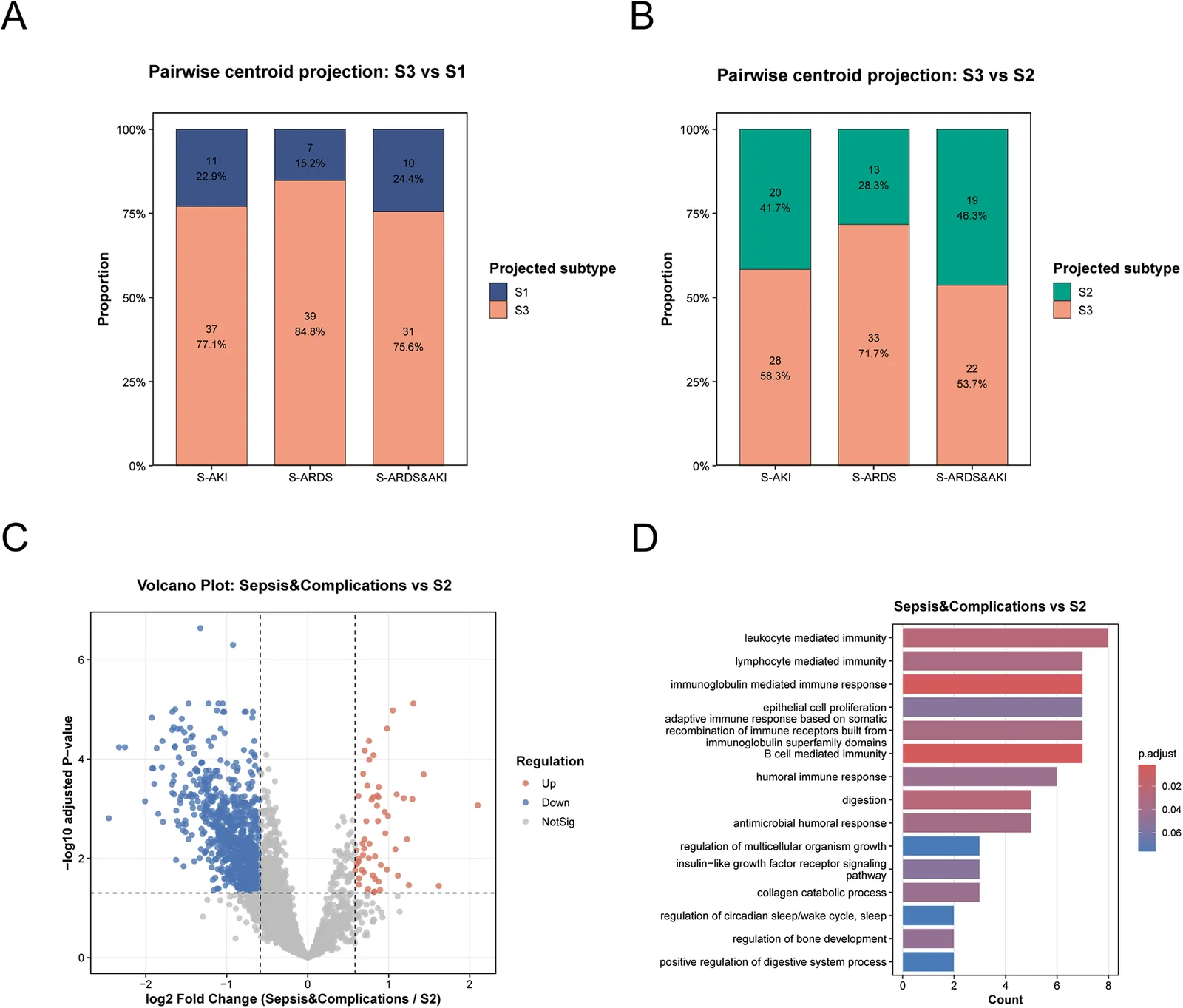
Molecular similarity between organ dysfunction-associated sepsis and the S3 EV-associated proteomic state. **(A,B)** Pairwise centroid projection of S-AKI, S-ARDS, and S-ARDS&AKI samples based on S3 vs S1 **(A)** and S3 vs S2 **(B)** pair-specific differentially abundant EV-associated proteins. Stacked bars indicate the proportions of samples assigned to each projected subtype. **(C)** Volcano plot of differentially expressed EV-associated proteins between Sepsis&Complications and S2. Sepsis&Complications included S-AKI, S-ARDS, and S-ARDS&AKI samples. **(D)** GO Biological Process enrichment analysis of proteins up-regulated in Sepsis&Complications compared with S2.

For pathway-level characterization, the three organ dysfunction groups were combined as the Sepsis&Complications group and compared with S2. Differential expression analysis identified EV-associated proteins with altered abundance in Sepsis&Complications relative to S2 (Figure 6C). GO Biological Process analysis of the up-regulated proteins identified several enriched processes, including leukocyte- and lymphocyte-mediated immunity, immunoglobulin-mediated immune responses, epithelial cell proliferation, adaptive immune responses, B-cell-mediated immunity, humoral and antimicrobial humoral responses, digestion, and insulin-like growth factor receptor signaling-related processes, among others (Figure 6D).

### 3.6 Adjusted and sensitivity analyses show reference-dependent S3-like similarity

After adjustment for APACHE II score, septic shock, and chronic-comorbidity burden, the three organ dysfunction groups had higher standardized S3-like projection margins than their corresponding S1 or S2 reference groups. Across the six comparisons, adjusted *β* coefficients ranged from 0.874 to 1.465, and all remained significant after Benjamini–Hochberg correction (all *q* < 0.001; Figure 7 and Supplementary Table S4).

**Figure 7:**
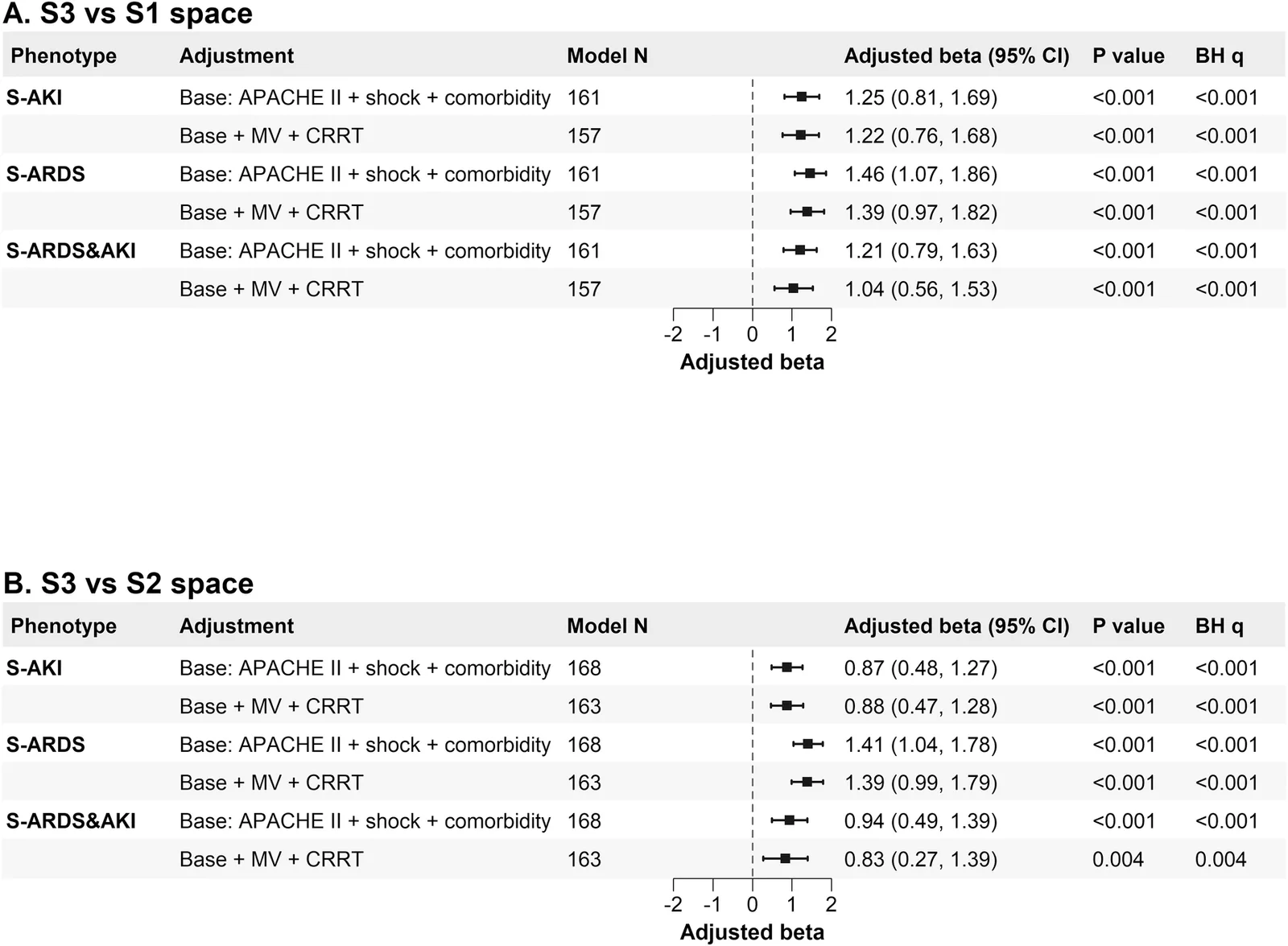
Adjusted associations between organ dysfunction phenotypes and S3-like proteomic similarity. Adjusted beta coefficients and 95% confidence intervals are shown for S-AKI, S-ARDS, and S-ARDS&AKI in the S3 vs S1 **(A)** and S3 vs S2 **(B)** projection spaces. The base model included APACHE II score, septic shock, and chronic-comorbidity burden; the organ-support model additionally included mechanical ventilation and continuous renal replacement therapy. *P* values were corrected using the Benjamini–Hochberg method.

Additional adjustment for invasive mechanical ventilation and CRRT recorded during the first 24 h after ICU admission produced limited changes in the estimates (adjusted *β* range, 0.835–1.393; all *q* ≤ 0.004). The direction of the associations was similar in sensitivity analyses using Spearman correlations, replacing APACHE II with SOFA, replacing septic shock with vasopressor use recorded during the first 24 h after ICU admission, or excluding the S-ARDS&AKI group (Supplementary Table S4).

When all Sepsis-only patients were used as the reference population, the associations were attenuated. None remained significant in the S3 vs S1 projection space, while only S-ARDS remained significant in the S3 vs S2 space after organ-support adjustment (adjusted *β* = 0.540, 95% CI, 0.209–0.870; *q* = 0.009). Thus, adjusted S3-like proteomic similarity was most consistent for S-ARDS, whereas evidence for S-AKI and S-ARDS&AKI depended on the subtype-specific reference comparison (Supplementary Figure S5 and Supplementary Table S4).

### 3.7 Derivation of a protein panel from proteins shared by S3 and the organ dysfunction groups

To derive a protein panel representing the proteomic features shared by S3 and the organ dysfunction groups, proteins with higher abundance in both S3 vs S2 and Sepsis&Complications vs S2 were intersected. This analysis identified 36 candidate proteins shared by the two comparisons (Figure 8A). Elastic-net logistic regression was fitted for the prespecified Sepsis&Complications vs S2 classification task. The full-cohort model retained 15 proteins: RNASE1, OIT3, IGHV3–38, RBP4, IGFBP4, CFD, IGLV1–40, KRTDAP, ATRN, CST3, IGLV1–36, IGHV1–18, APOH, ACKR1, and CCL14 (Figure 8B). The resulting full-cohort classifier achieved an apparent AUROC of 0.871 (95% CI, 0.808–0.934; Figure 8C). Model-derived scores were lowest in S2, intermediate in S1, and higher in S3 and Sepsis&Complications (Figure 8D). A model based on the same panel distinguished S3 from S2 with an apparent AUROC of 0.958 (95% CI, 0.900–1.000), while one vs rest discrimination among the Sepsis-only subtypes was highest for S3 (AUROC = 0.938; Supplementary Figure S6).

**Figure 8:**
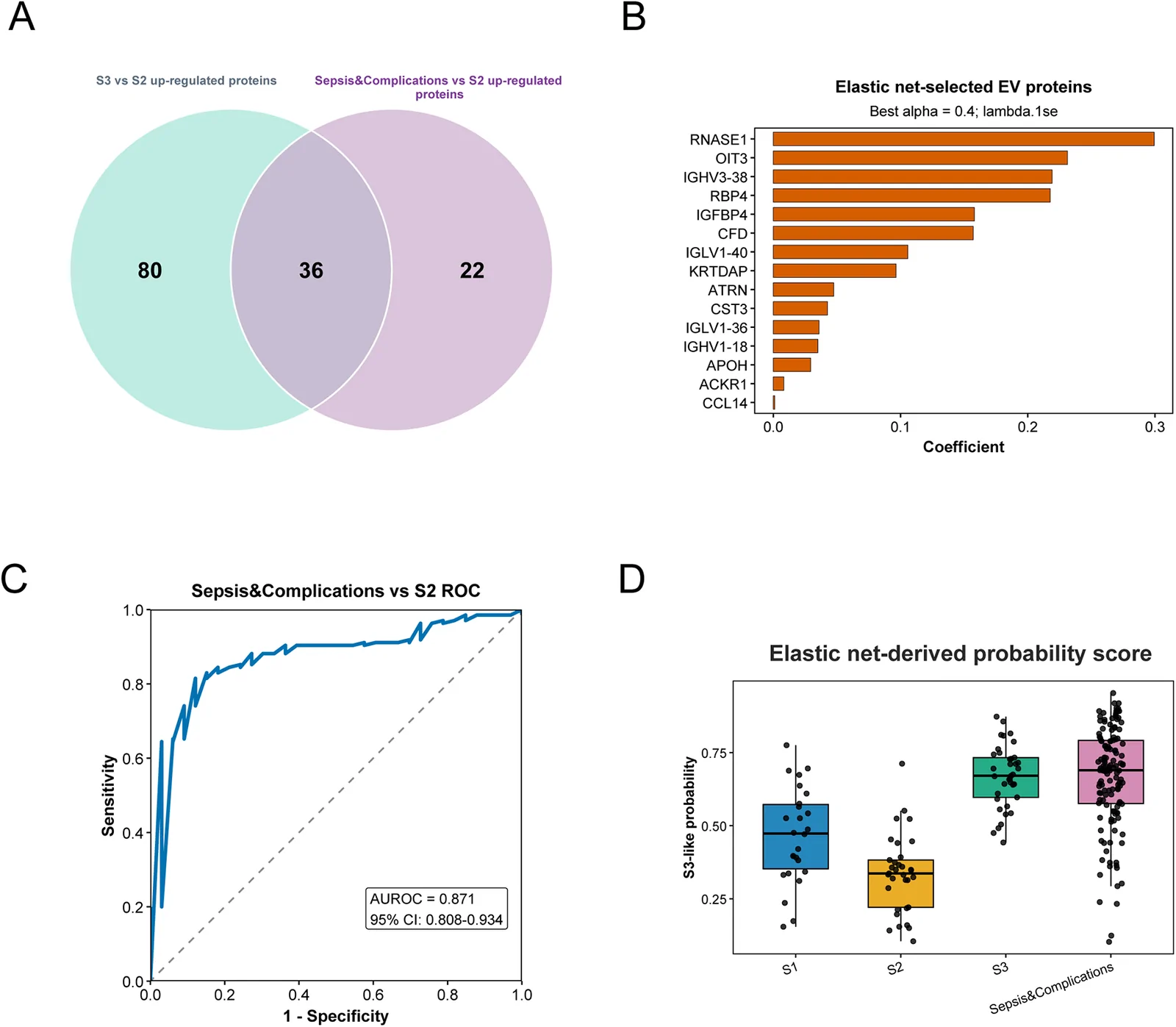
An internally selected 15-protein EV-enriched fraction-associated panel and its apparent full-cohort performance. **(A)** Venn diagram showing the overlap between EV-associated proteins up-regulated in S3 vs S2 and those up-regulated in Sepsis&Complications vs S2. Thirty-six shared up-regulated proteins were used as candidate proteins for elastic net modelling. **(B)** Coefficient plot of the 15 proteins retained in the full-cohort exploratory elastic net model. **(C)** Apparent full-cohort receiver operating characteristic curve for distinguishing Sepsis&Complications from S2. **(D)** Distribution of the 15-protein panel-derived probability score across S1, S2, S3, and Sepsis&Complications groups.

To assess the robustness of these findings to missing-value handling, KNN imputation was applied to the same missing entries addressed by the primary strategy (19.55% of all protein–sample measurements); complete protein- and group-specific missingness statistics are provided in Supplementary Data S1. The KNN analysis reproduced 86 of 96 NMF assignments (89.6%; adjusted Rand index, 0.707); all 36 candidates retained higher abundance in both comparisons, and 31—including 13 of the 15 panel proteins—remained differentially expressed according to the original criteria (Supplementary Table S5).

### 3.8 Repeated nested internal validation of the downstream supervised candidate-selection and modelling workflow

Repeated nested validation of the downstream supervised workflow was performed conditional on the previously derived NMF subtype labels. The primary Sepsis&Complications versus S2 analysis included 168 patients (135 versus 33). Across 100 repetitions of five-fold nested cross-validation, the mean AUROC was 0.847 (95% CI, 0.777–0.902), and the mean PR-AUC was 0.955 (95% CI, 0.928–0.976). The training-derived decision threshold had a median of 0.784 (IQR, 0.768–0.798) across the 500 outer folds. Patient-level consensus classification yielded 29 true negatives, 4 false positives, 31 false negatives, and 104 true positives, corresponding to a sensitivity of 0.770, a specificity of 0.879, a positive predictive value of 0.963, and a negative predictive value of 0.483 (Figures 9A, B, E; Supplementary Figure S7A; Supplementary Table S6). For the patient-aggregated outer out-of-fold probabilities, within-fold recalibration changed the calibration intercept from 0.002 to −0.004 and the slope from 1.725 to 0.999, while the Brier score decreased from 0.125 to 0.120 (Figure 9C; Supplementary Table S6). Decision-curve analysis showed greater net benefit than the treat-all and treat-none strategies across threshold probabilities of 0.44–0.95 (Figure 9F; Supplementary Table S6).

**Figure 9:**
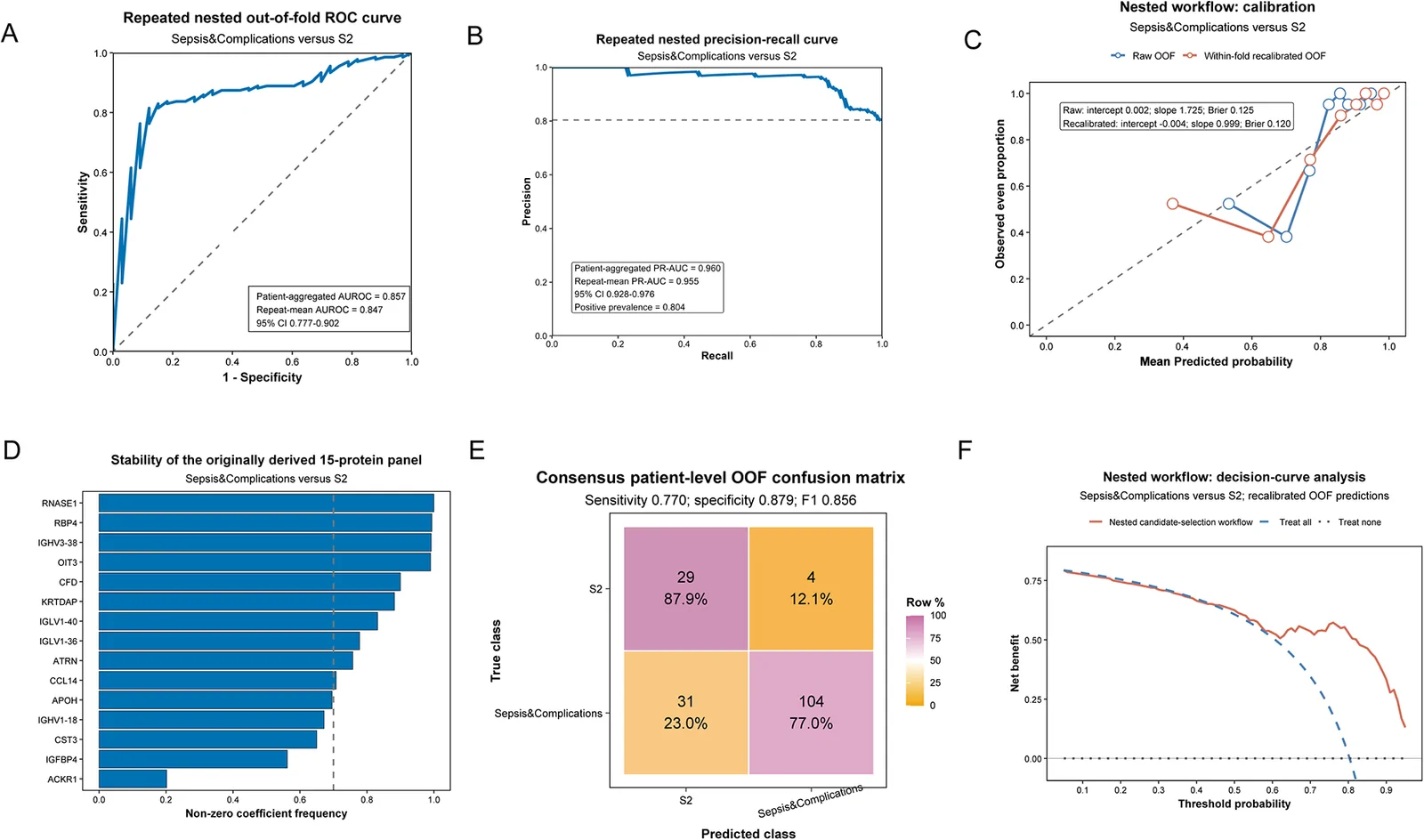
Repeated nested internal validation of the downstream supervised proteomic modelling workflow. **(A)** Patient-aggregated outer out-of-fold ROC curve for Sepsis&Complications versus S2. **(B)** Corresponding precision–recall curve. **(C)** Calibration of raw and within-fold recalibrated patient-aggregated outer out-of-fold probabilities; points represent eight equal-frequency groups. **(D)** Non-zero coefficient frequencies of the originally reported 15 proteins across 500 outer folds. **(E)** Patient-level consensus confusion matrix. **(F)** Decision-curve analysis of the recalibrated outer out-of-fold probabilities compared with treat-all and treat-none strategies.

The secondary S3 versus S2 analysis included 37 S3 and 33 S2 patients. The mean AUROC was 0.956 (95% CI, 0.912–0.989), and the mean PR-AUC was 0.951 (95% CI, 0.904–0.989). Consensus classification yielded 29 true negatives, 4 false positives, 2 false negatives, and 35 true positives. The median training-derived threshold was 0.557 (IQR, 0.502–0.618) and showed greater variability than that of the primary analysis (Supplementary Figure S7B; Supplementary Table S6).

Bootstrap optimism correction yielded corrected AUROC and PR-AUC estimates of 0.859 and 0.960, respectively, for Sepsis&Complications versus S2, and 0.980 and 0.979 for the separately fitted S3 versus S2 workflow (Supplementary Figures S7C, D; Supplementary Table S6). Protein-selection frequencies and the numbers of candidate and selected proteins varied across resamples, indicating variation in the exact feature composition (Figure 9D; Supplementary Figure S8; Supplementary Table S7). In the supportive fixed-panel analysis, the 15-protein models yielded mean AUROCs of 0.887 for Sepsis&Complications versus S2 and 0.961 for S3 versus S2 (Supplementary Table S6). Overall, the downstream workflows retained discriminatory information across internal resampling, although all performance estimates remained conditional on the NMF subtype labels derived from the full Sepsis-only cohort.

## 4 Discussion

In the present study, NMF analysis of serum EV-enriched proteomic profiles identified three molecular subtypes within the Sepsis-only cohort. S1 showed comparatively less pronounced inflammatory and adverse outcome-related features, whereas these features were more evident in S2 and S3. Notably, S2 and S3 overlapped in APACHE II and SOFA scores, indicating that their molecular separation was not simply a reflection of conventional clinical severity. This disconnect between bedside phenotype and molecular organization is consistent with previous plasma proteomic and transcriptomic studies of sepsis (7–9, 27, 28). Mi et al. identified proteomic subphenotypes with distinct host-response and outcome associations, while Bracht et al. separated immunologically different subtypes from a small, highly severe cluster interpreted as a possible terminal phase of illness (7, 9). Our findings extend this evidence of heterogeneity to serum EV-enriched preparations obtained from patients without ARDS or AKI at sampling. We therefore use S1, S2, and S3 as neutral labels for the proteomic patterns observed in this cohort, without equating them with previously reported endotypes or successive stages of sepsis. Because the purpose of NMF subtyping was to explore molecular heterogeneity within sepsis, we examined whether the stable k = 2 structure contained a biologically informative higher-resolution subdivision. Although k = 2 was more stable, k = 3 further resolved distinct cellular/metabolic and humoral immune/complement–coagulation patterns, with the core S3 biology preserved at k = 2. We therefore regard k = 3 as an exploratory higher-resolution representation that requires external confirmation.

The clearest biological contrast emerged between S2 and S3. S2 combined leukocyte migration, phagocytosis, and endocytosis with oxidative phosphorylation, mTOR-related signaling, and the unfolded protein response. Viewed together, these pathways suggest coupling of cellular immune activity with energetic demand and protein-folding stress. This interpretation is compatible with observations that monocytes obtained during septic shock undergo simultaneous changes in inflammatory competence and energy metabolism, including disruption of tricarboxylic acid-cycle and oxidative-phosphorylation programs during the acute phase (29). Experimental work in LPS-stimulated human monocytes has also shown that mTOR-dependent metabolic regulation can restrain, rather than uniformly amplify, inflammatory cytokine and tissue-factor expression (30). S3 showed a different configuration in which immunoglobulin- and B-cell-related proteins occurred alongside complement, coagulation, hemostatic, and acute-phase proteins. The immune, endothelial, complement, and coagulation systems interact closely during the intravascular response to infection, but dysregulation of these interactions can also contribute to microvascular injury and organ dysfunction (31, 32). Related combinations of adaptive immune, acute inflammatory, complement, and coagulation features have appeared across previously reported sepsis molecular subtypes (7–9), and plasma EV proteomics has identified linked modules involving innate immunity, complement/coagulation, platelet activation, endothelial activation, and organ injury (33). The S2 and S3 distinction therefore points to different configurations of the host response: one centered on cellular immune and metabolic stress and the other on a humoral, complement/coagulation, and vascular-inflammatory program.

The extension of the S3 pattern to established organ dysfunction was partial and reference-dependent. Although the organ dysfunction groups showed group-level shifts toward S3 after clinical adjustment, overlap between S2 and S3 limited individual separation, and most associations weakened when the entire Sepsis-only cohort was used as the reference. The shared signal therefore appeared to arise mainly from the contrast with S2 rather than from a general molecular boundary between sepsis with and without organ dysfunction. The lack of a stronger association in S-ARDS&AKI also argues against interpreting S3-like similarity as a cumulative measure of organ-failure severity. The most consistent relationship was observed for S-ARDS. Plasma and bronchoalveolar lavage fluid proteomics have identified complement/coagulation and compartment-specific immune processes in ARDS (34), while studies of sepsis-related ARDS have linked endothelial or circulating EVs and their cargoes to vascular injury and lung dysfunction (35–37). These findings provide biological context but do not directly validate the present serum EV-enriched signature. Overall, the projection results indicate contemporaneous sharing of circulating proteomic features, most consistently in S-ARDS, rather than a common ARDS/AKI endotype or prediction of subsequent organ dysfunction.

The full-cohort elastic-net fit retained 15 proteins as a compact representation of the pattern shared by S3 and the organ dysfunction groups relative to S2. Its biological interpretation rests on the combined profile rather than any single protein. The panel included immunoglobulin variable-region proteins and proteins related to complement, coagulation, vascular responses, and tissue injury. Among these, CFD participates in alternative complement pathway regulation, whereas APOH interacts with phospholipid-dependent complement and coagulation processes (38, 39). CST3, IGFBP4, and CFD have also been reported in circulating proteomic studies of SA-AKI, while urinary CCL14 has been associated with persistent severe AKI (40–44). However, differences in biospecimen, sampling time, and clinical endpoint preclude direct equivalence with the present serum EV-enriched measurements. Accordingly, the panel represents a focused candidate set reflecting the molecular overlap between S3 and established organ dysfunction rather than an organ-specific biomarker set.

The primary supervised comparison was imbalanced, with Sepsis&Complications accounting for 80.4% of the analysis set. Although the model showed a high positive predictive value, its low negative predictive value indicates that it should not be used to rule out organ dysfunction. The panel is therefore considered a hypothesis-generating molecular signature rather than a stand-alone clinical test. Following longitudinal assessment, external validation, and prevalence-specific recalibration, it could be evaluated as an adjunctive prioritization tool for identifying patients with an S3-like molecular pattern rather than as a rule-out test.

These biological interpretations must also account for the EV-enriched nature of the analyzed material. Classical EV-associated markers, vesicle-like structures, the observed particle-size distribution, and a broad EV-associated protein repertoire support enrichment of EV-containing material but do not establish the vesicular localization of every quantified protein, consistent with MISEV2023 (45). Proteomic profiles of serum EV-enriched preparations may vary according to the enrichment method, underscoring the importance of interpreting the present findings within the context of the serum matrix (46, 47). Blood EV isolation is particularly challenging because soluble protein complexes and lipoprotein particles are substantially more abundant than EVs and overlap with them in physical properties (48, 49). Circulating proteins may also associate with the EV surface as a protein corona containing apolipoproteins, immunoglobulins, complement proteins, and fibrinogen (50). Against this background, comparison with a recent 25-protein plasma signature distinguishing sepsis from non-infectious systemic inflammatory response syndrome identified six shared proteins—APOH, CFB, IGFALS, ORM1, PPBP, and SERPINA3—among those elevated in S3 (51). Of these, only APOH was retained in our 15-protein panel, suggesting convergence in complement, coagulation, and acute-phase biology without simple recapitulation of the reported plasma signature. Although albumin, apolipoproteins, and intracellular-compartment-associated proteins were detected in our preparations, only ALB was significantly higher in S3 than in S2; the other evaluated co-isolate indicators did not show a generalized increase (Supplementary Figure S9; Supplementary Tables S8 and S9). Because the cellular origin of these proteins was not directly assessed, associations with platelet, endothelial, or leukocyte biology remain pathway-level interpretations rather than evidence of vesicle origin. Nevertheless, APOH, CFD, and immunoglobulin variable-region chains could represent EV-surface or corona-associated proteins, or non-vesicular protein and lipoprotein complexes retained during enrichment, rather than exclusively intravesicular cargo. Accordingly, we interpret these proteins as features of the serum EV-enriched fraction without assigning them specifically to the intravesicular compartment. Because paired particle counts and protein measurements were unavailable, a reliable particle-to-protein ratio could not be calculated retrospectively.

The main limitations of this study arise from its single-center, cross-sectional design. No longitudinal or external cohort was available to determine whether an S3-like state preceded the subsequent development of ARDS or AKI. ARDS and AKI status was assessed at blood sampling, and subsequent development was not systematically recorded in the Sepsis-only cohort; the findings therefore reflect contemporaneous molecular similarity rather than progression or prediction. Exact intervals from symptom onset or antibiotic initiation to sampling and pre-analytical processing were unavailable for covariate adjustment. Non-differential variation in these intervals would be expected primarily to increase within-group variability and attenuate the observed associations, whereas systematic differences between groups could bias the similarity estimates in either direction; the magnitude of these effects could not be quantified retrospectively. Restricting subtype discovery to patients without ARDS or AKI reduced the influence of established complication phenotypes but may have underrepresented severe or rapidly evolving molecular states, thereby limiting generalizability. In addition, the NMF labels were derived from the full Sepsis-only cohort, S2 served as both a subtype-discovery product and the common reference in the candidate-generating contrasts, and the 15-protein panel was selected and evaluated within the same cohort. Consequently, nested validation did not eliminate this structural circularity, and the resulting internal performance estimates may remain substantially optimistic and should be considered hypothesis-generating. Future multicenter studies should use serial sampling before the onset of ARDS or AKI, prospectively record clinical and pre-analytical sampling intervals, and externally validate a prespecified protein panel.

## 5 Conclusions

Serum EV-enriched proteomics revealed distinct host-response subtypes within the Sepsis-only group and identified molecular features shared between one subtype and established organ dysfunction. These findings advance the molecular characterization of sepsis heterogeneity and define a focused set of candidate proteins for further investigation. Because the relationships were observed at the time of sampling, they indicate contemporaneous molecular similarity rather than prediction of subsequent organ dysfunction. Future longitudinal and external validation will help determine their relevance to molecular stratification in sepsis.

## Data Availability

The mass spectrometry proteomics data have been deposited to the ProteomeXchange Consortium via the iProX partner repository with the dataset identifier PXD083045 (iProX accession: IPX0018701000). The dataset can be accessed at: https://www.iprox.cn/page/project.html?id=IPX0018701000.
