# Supplementary material for: Serum EV-enriched proteomics reveals host-response subtypes and molecular similarity to organ dysfunction phenotypes in sepsis
Source: Front Med (Lausanne). 2026 Oct 7;13:1896167. doi: 10.3389/fmed.2026.1896167 (PMC13683613; doi:10.3389/fmed.2026.1896167)
Supplement: Supplementary file 1 [file Image1.pdf]

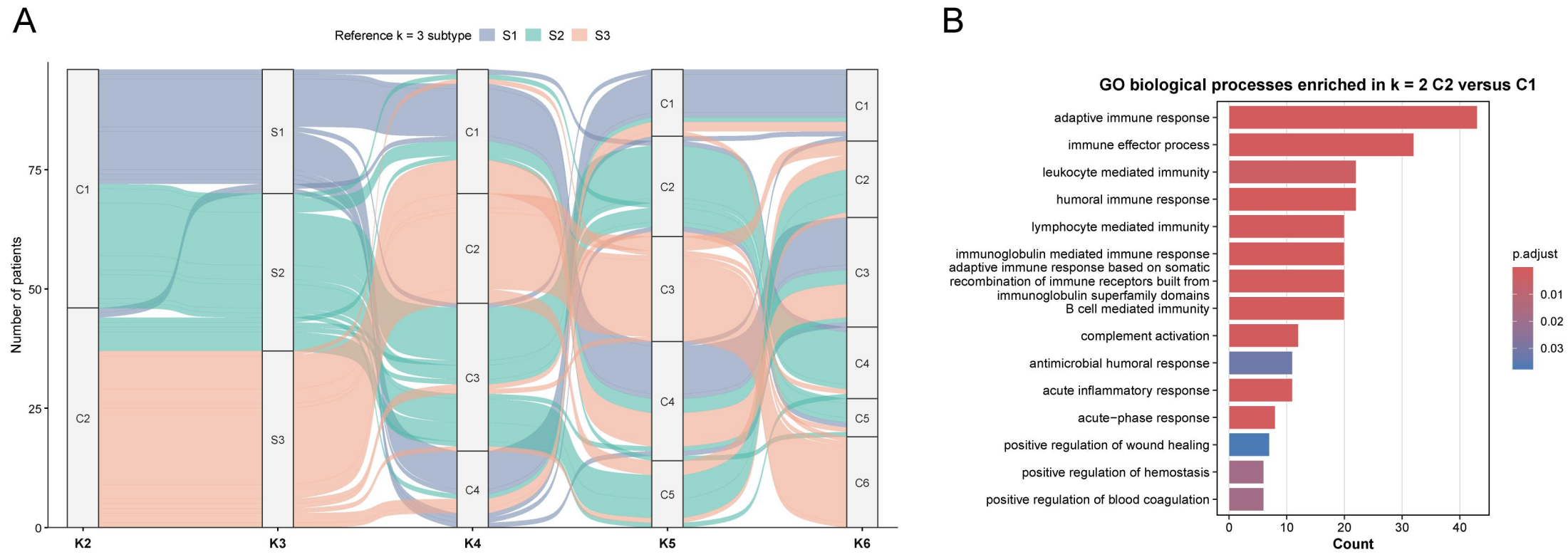

Supplementary Figure S1. NMF rank sensitivity analysis. (A) Patient membership across NMF ranks (k = 2–6), colored by the (k = 3) subtype. (B) GO biological processes enriched among proteins with higher abundance in the (k = 2) C2 cluster than in C1. Bar length indicates protein count, and color indicates the adjusted *P*-value.

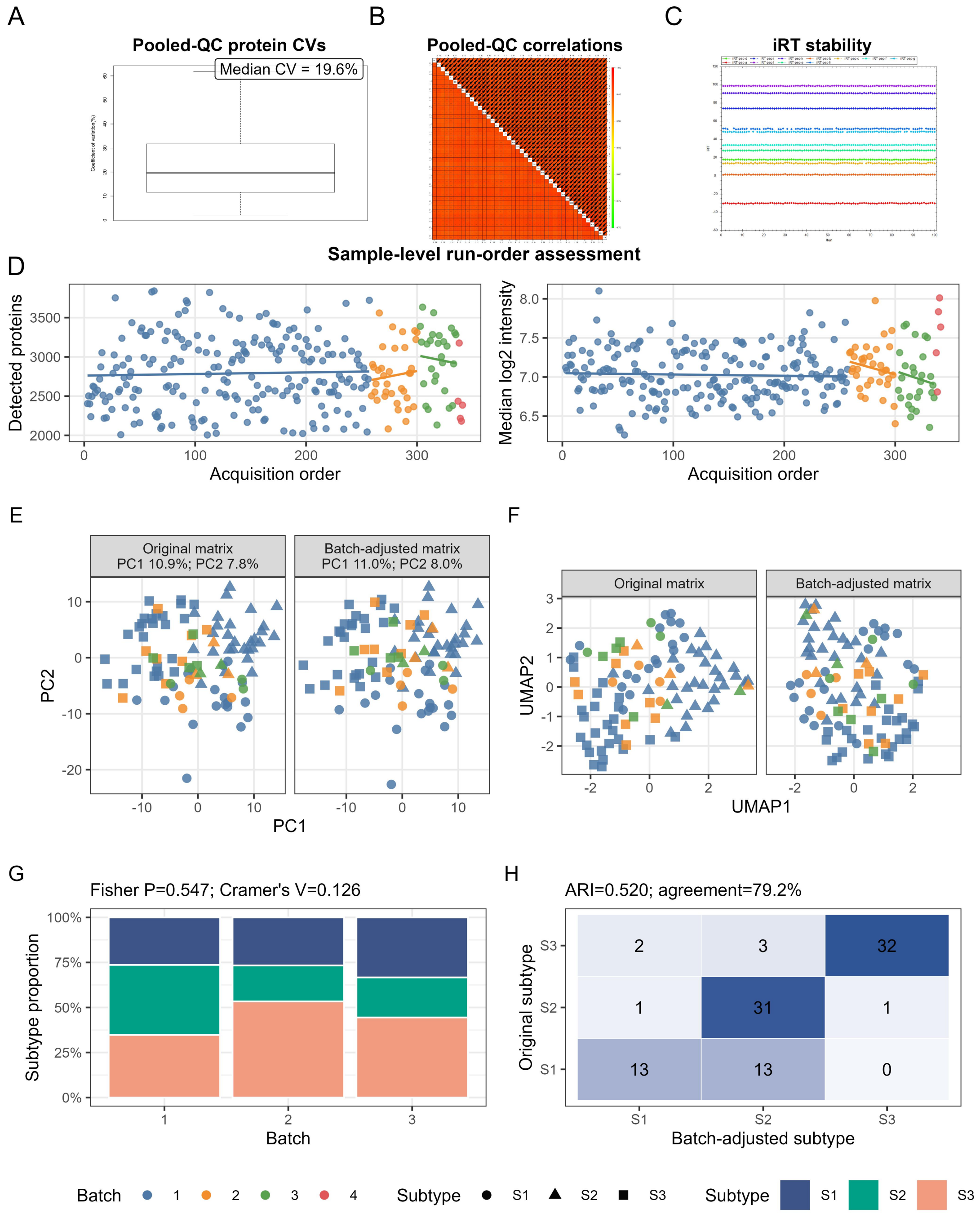

Supplementary Figure S2. Batch and quality-control assessment.

(A – C) Pooled-QC reproducibility and iRT stability. (D) Sample-level metrics across the acquisition sequence. (E – F) PCA and UMAP before and after batch adjustment. (G) Subtype distribution across batches. (H) Concordance between the original and batch-adjusted NMF assignments.

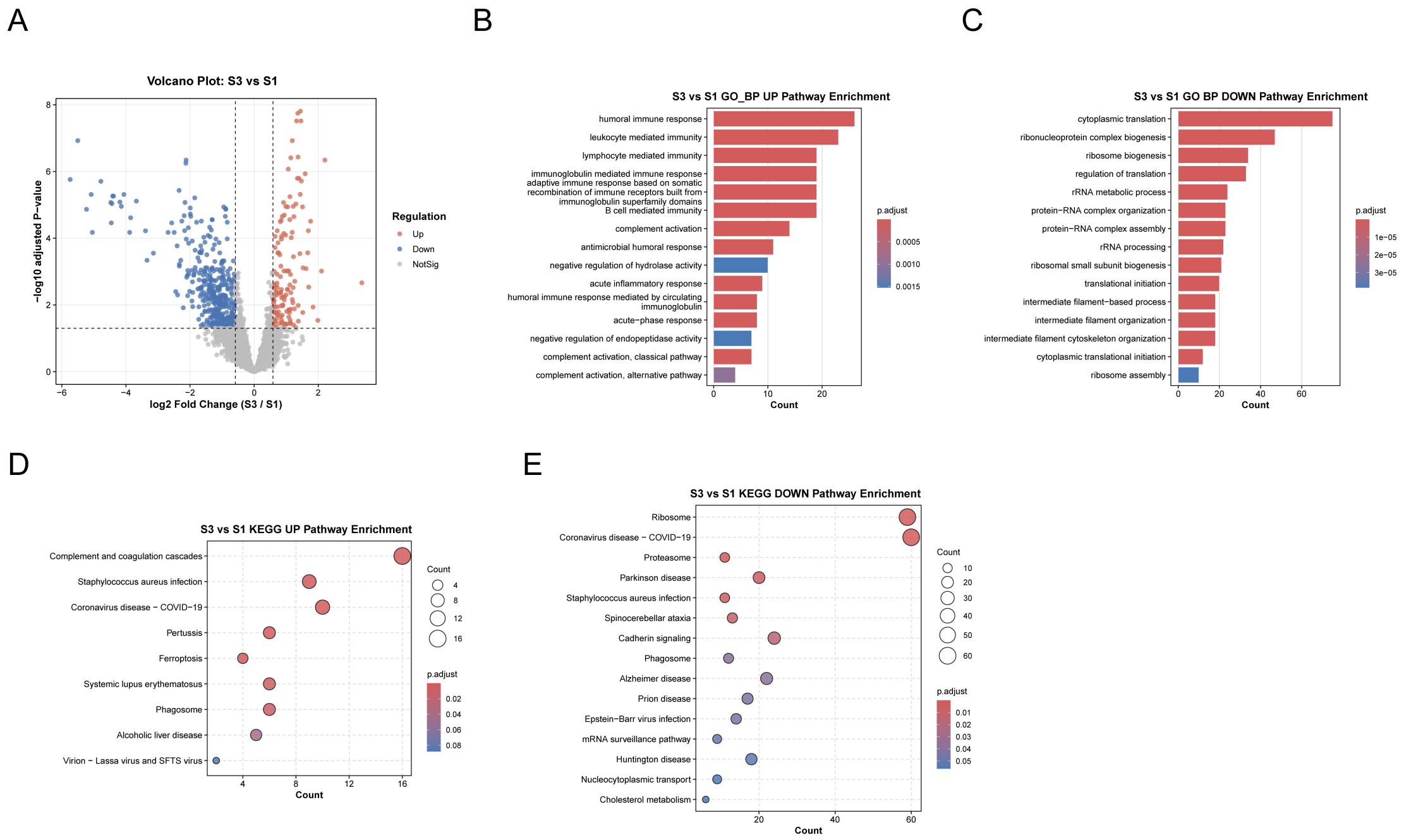

Supplementary Figure S3. Differential EV-associated proteins and pathway enrichment analysis between S3 and S1.

(A) Volcano plot showing differentially expressed EV-associated proteins between S3 and S1. Proteins with higher levels in S3 are shown in red, proteins with lower levels in S3 are shown in blue, and non-significant proteins are shown in gray.

(B) GO Biological Process enrichment analysis of proteins up-regulated in S3 compared with S1.

(C) GO Biological Process enrichment analysis of proteins down-regulated in S3 compared with S1.

(D) KEGG pathway enrichment analysis of proteins up-regulated in S3 compared with S1.

(E) KEGG pathway enrichment analysis of proteins down-regulated in S3 compared with S1.

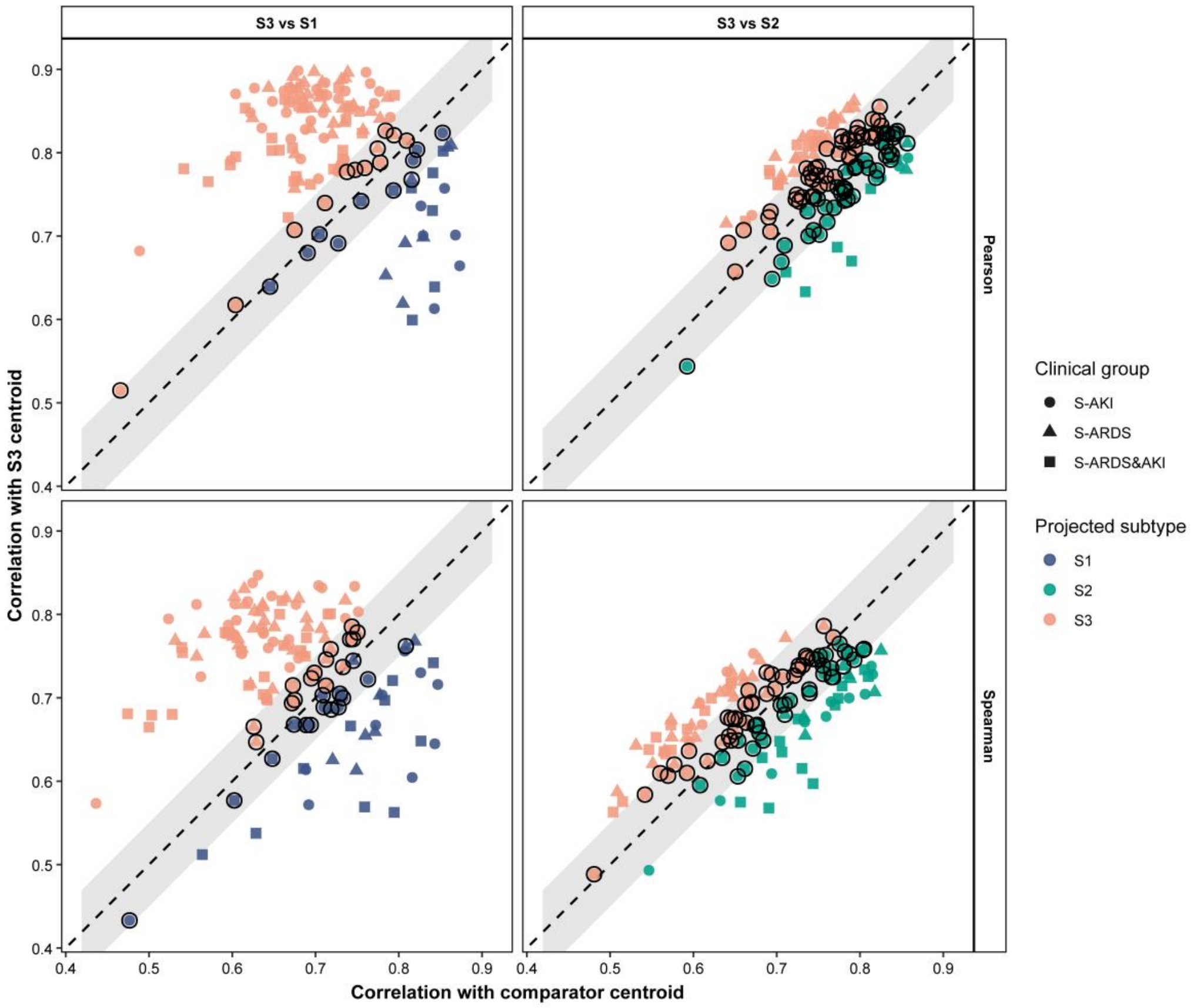

Supplementary Figure S4. Sample-level Pearson and Spearman correlation distributions in the pairwise centroid projection analysis.

A. S3 vs S1 space

Phenotype	Model N	Adjusted beta (95% CI)	P value	BH q
S-AKI	231	0.11 (-0.23, 0.45)	0.523	0.829
S-ARDS	231	0.33 (0.05, 0.62)	0.023	0.068
S-ARDS&AKI	231	0.06 (-0.25, 0.37)	0.717	0.829

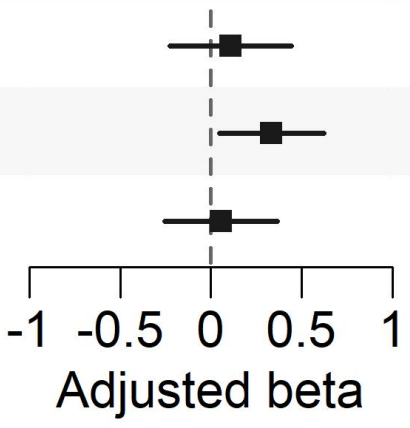

B. S3 vs S2 space

Phenotype	Model N	Adjusted beta (95% CI)	P value	BH q
S-AKI	231	0.04 (-0.32, 0.40)	0.829	0.829
S-ARDS	231	0.58 (0.25, 0.91)	<0.001	0.004
S-ARDS&AKI	231	0.07 (-0.34, 0.48)	0.744	0.829

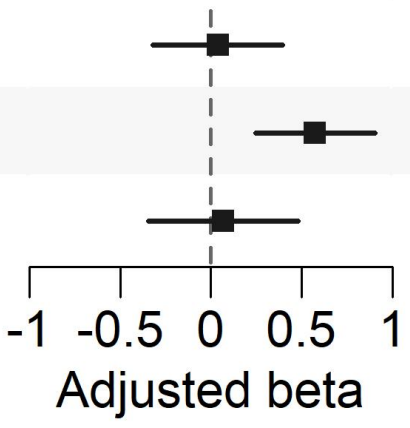

Supplementary Figure S5. Sensitivity analysis using the entire Sepsis-only cohort as the reference population.

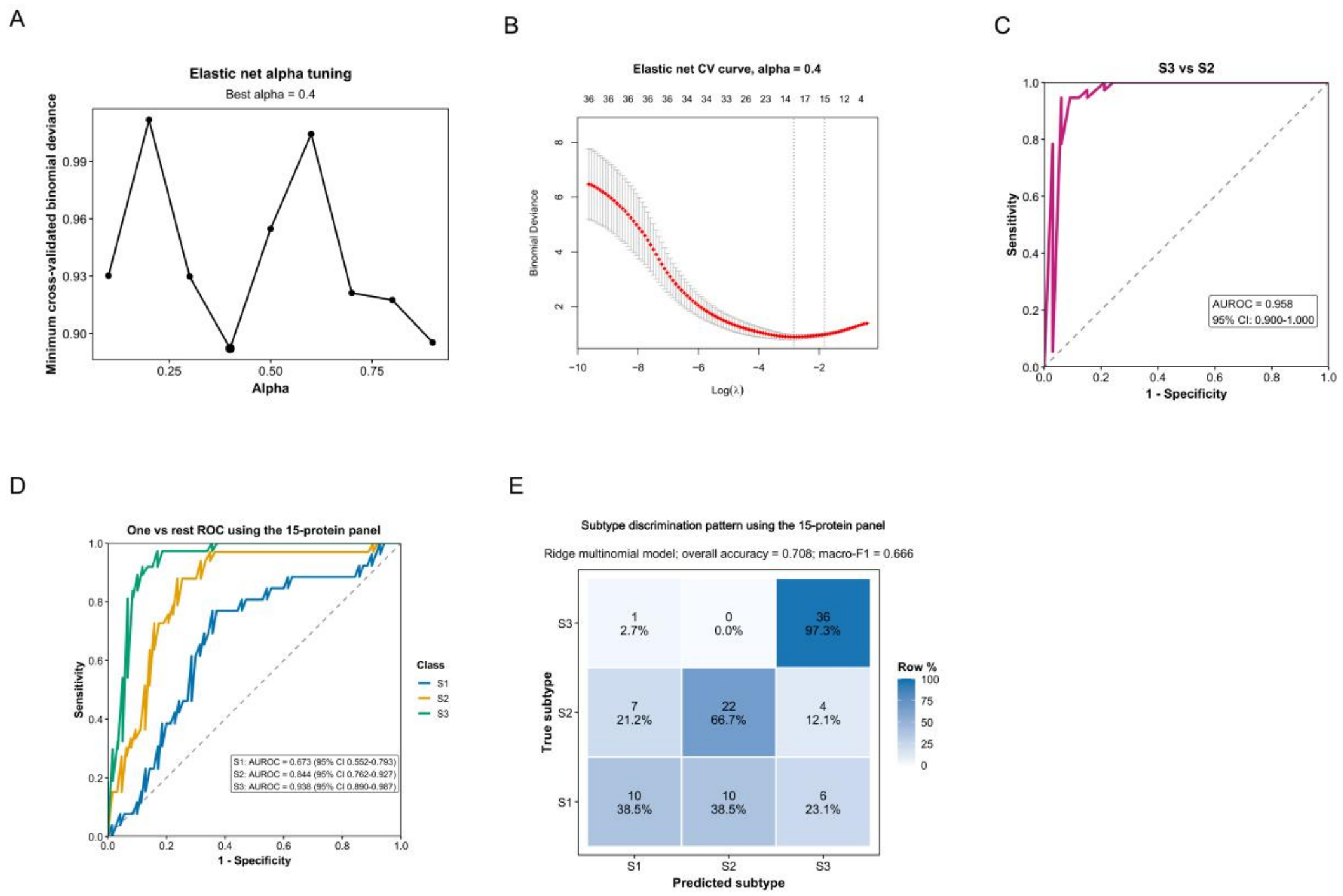

Supplementary Figure S6. Elastic net model diagnostics and apparent performance of the internally selected 15-protein panel.

(A) Alpha tuning for elastic net logistic regression. The optimal alpha was 0.4 based on minimum cross-validated binomial deviance.

(B) Cross-validation curve for lambda selection at alpha = 0.4. Vertical dashed lines indicate lambda values selected during model tuning.

(C) Receiver operating characteristic curve evaluating the ability of the 15-protein panel to distinguish S3 from S2 among Sepsis-only patients.

(D) One-vs-rest receiver operating characteristic curves evaluating exploratory discrimination of S1, S2, and S3 among Sepsis-only patients using the 15-protein panel.

(E) Confusion matrix from the fixed-panel ridge multinomial model showing subtype discrimination patterns among S1, S2, and S3.

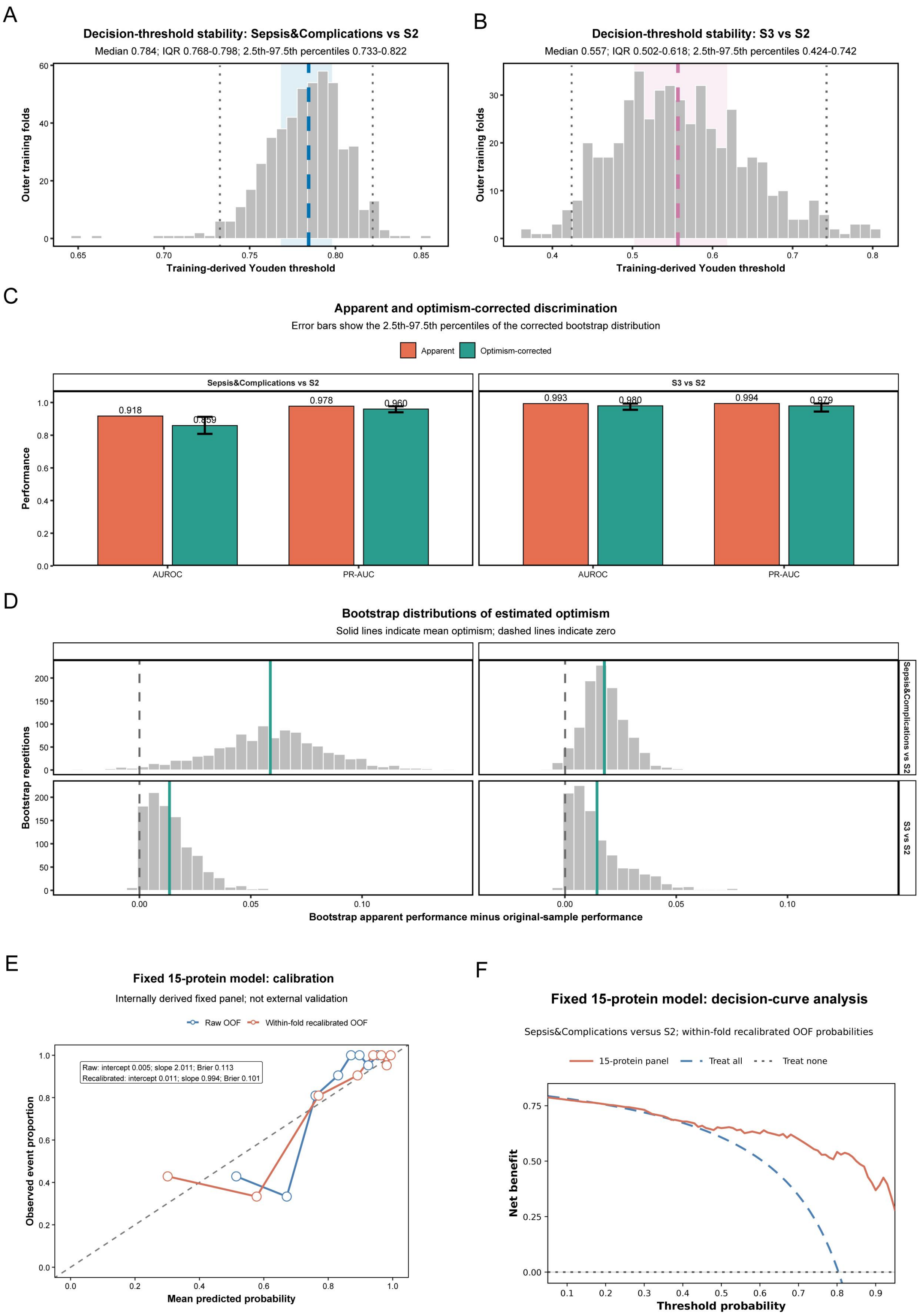

Supplementary Figure S7. Additional internal-validation diagnostics for the supervised modelling analyses.

(A, B) Stability of training-derived decision thresholds. (C, D) Apparent and optimism-corrected discrimination. (E) Calibration of the fixed 15-protein model before and after within-fold recalibration. (F) Decision-curve analysis of the recalibrated fixed 15-protein model.

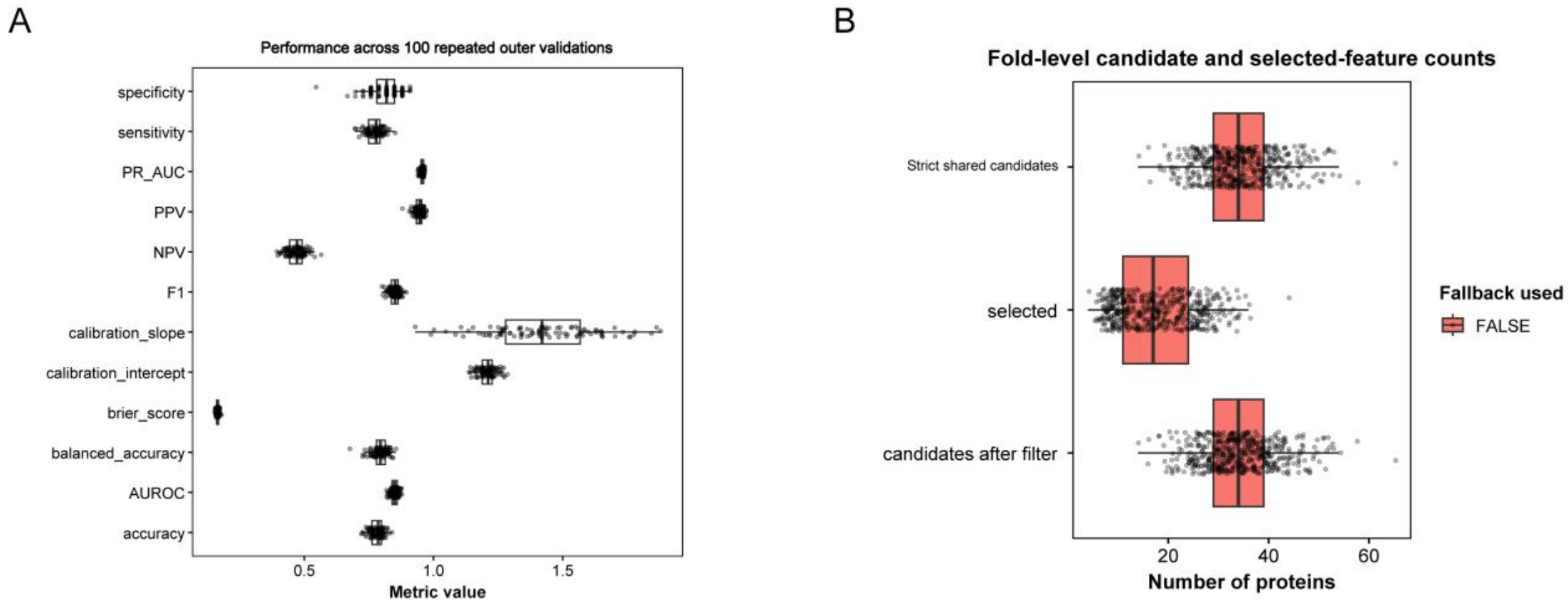

Supplementary Figure S8. Repeat- and fold-level stability diagnostics from repeated nested internal validation.

(A)Performance metrics across 100 repeated outer cross-validation runs. (B) Numbers of candidate and selected proteins across 500 outer folds.

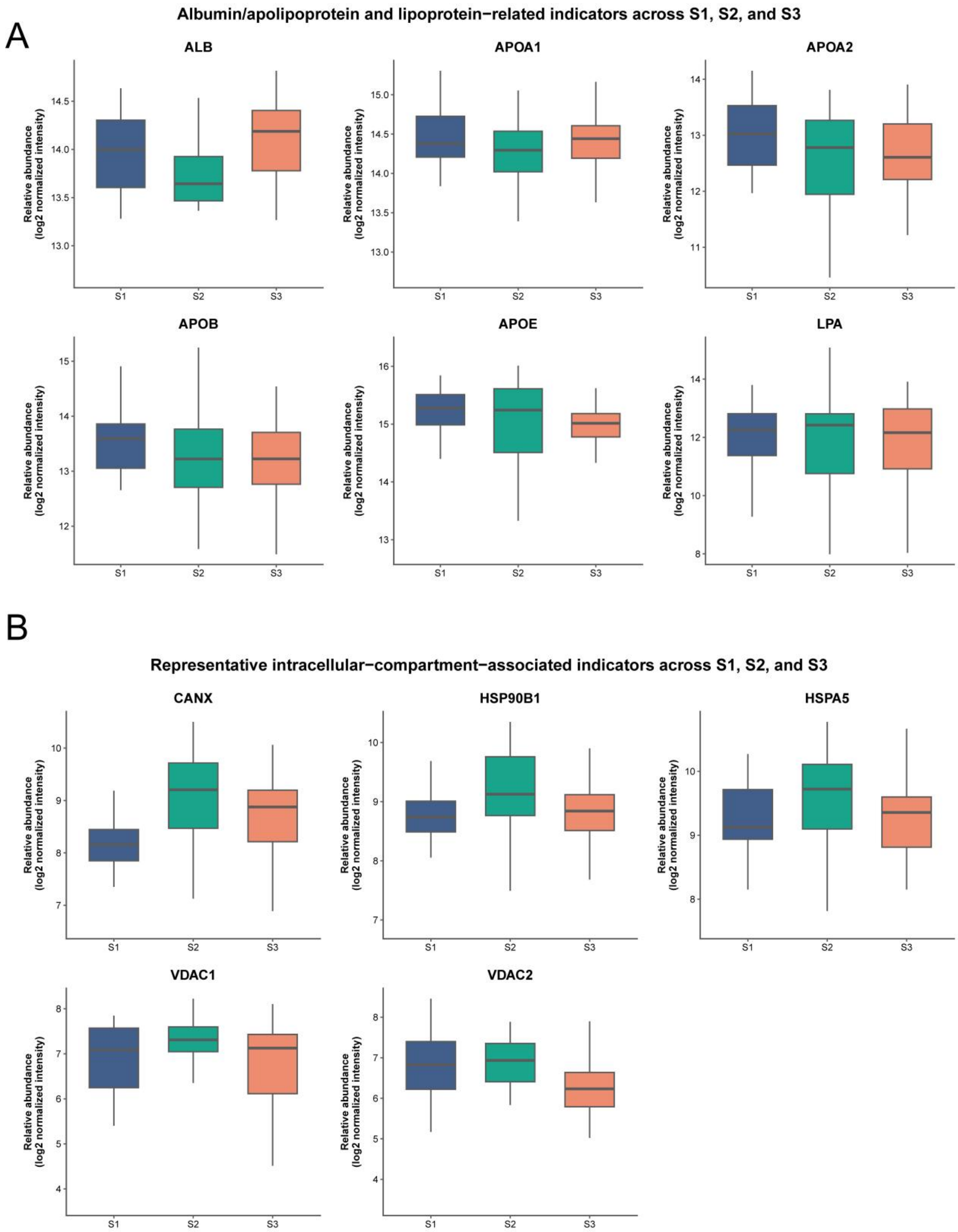

Supplementary Figure S9. Proteomics-based assessment of representative co-isolate indicators across EV-enriched proteomic subtypes.

(A) Relative abundances of albumin/apolipoprotein and lipoprotein-related indicators across S1 – S3. (B) Relative abundances of intracellular-compartment-associated indicators. Boxes show medians and interquartile ranges.
