# Supplementary material for: Serum EV-enriched proteomics reveals host-response subtypes and molecular similarity to organ dysfunction phenotypes in sepsis
Source: Front Med (Lausanne). 2026 Oct 7;13:1896167. doi: 10.3389/fmed.2026.1896167 (PMC13683613; doi:10.3389/fmed.2026.1896167)
Supplement: Supplementary file 2 [file Table1.docx]

**Supplementary Table S1. Quantitative evaluation of NMF factorization rank and cross-rank stability.**

**A. Rank-selection and patient-resampling stability metrics**

| **k** | **NMF runs** | **Cophenetic coefficient** | **Dispersion** | **Mean consensus silhouette** | **RSS** | **CDF area** | **Relative delta area** | **Cluster size range** | **Resampling ARI, median (IQR)** |
| --- | --- | --- | --- | --- | --- | --- | --- | --- | --- |
| 2 | 100 | 0.989 | 0.859 | 0.9492 | 1948.49 | 0.509 | — | 46–50 | 0.751 (0.705–0.846) |
| 3 | 100 | 0.980 | 0.842 | 0.8965 | 1779.18 | 0.672 | 0.322 | 26–37 | 0.614 (0.506–0.747) |
| 4 | 100 | 0.970 | 0.821 | 0.8441 | 1636.20 | 0.750 | 0.115 | 16–31 | — |
| 5 | 100 | 0.965 | 0.799 | 0.7829 | 1519.66 | 0.803 | 0.070 | 14–25 | — |
| 6 | 100 | 0.971 | 0.832 | 0.7866 | 1420.31 | 0.825 | 0.028 | 8–23 | — |

Relative delta area was calculated against the preceding rank and is therefore not defined for k = 2. Patient-resampling stability was assessed for k = 2 and k = 3 using 100 repetitions, each sampling 80% of patients (76 of 96) without replacement and fitting NMF with 10 runs. ARI, adjusted Rand index; CDF, cumulative distribution function; IQR, interquartile range; NMF, non-negative matrix factorization; RSS, residual sum of squares.

**B. Preservation of the reference k = 3 S3 membership across factorization ranks**

| **k** | **Best-overlapping cluster** | **Reference S3, n** | **Comparison cluster, n** | **Overlap, n** | **S3 retention, %** | **Cluster purity for S3, %** | **Jaccard similarity** | **ARI versus k = 3** |
| --- | --- | --- | --- | --- | --- | --- | --- | --- |
| 2 | C2 | 37 | 46 | 37 | 100.0 | 80.4 | 0.804 | 0.472 |
| 3 | C3 | 37 | 37 | 37 | 100.0 | 100.0 | 1.000 | 1.000 |
| 4 | C2 | 37 | 23 | 23 | 62.2 | 100.0 | 0.622 | 0.424 |
| 5 | C3 | 37 | 22 | 22 | 59.5 | 100.0 | 0.595 | 0.296 |
| 6 | C6 | 37 | 19 | 19 | 51.4 | 100.0 | 0.514 | 0.248 |

Cluster identifiers are rank-specific and do not imply equivalence of equally numbered clusters across ranks. For each rank, the best-overlapping cluster was the cluster containing the largest number of members of the reference k = 3 S3 subtype (n = 37). S3 retention was calculated as the overlap divided by 37; cluster purity was calculated as the overlap divided by the size of the best-overlapping cluster. At k = 3, C3 denotes the raw NMF cluster corresponding to the displayed S3 subtype. ARI, adjusted Rand index.

**Supplementary Table S2. Acquisition-batch assessment and sensitivity of the NMF subtype structure.**

**A. Acquisition-order effects**

| **Metric** | **Scope** | **Spearman rho** | ***P* value** | **BH-adjusted *P* value** |
| --- | --- | --- | --- | --- |
| Detected proteins | All retained samples | 0.063 | 0.297 | 0.297 |
| Median log2 intensity | All retained samples | 0.018 | 0.770 | 0.770 |
| Detected proteins | Within Batches 1-3 | -0.057 to 0.050 | 0.470-0.790 | >=0.790 |
| Median log2 intensity | Within Batches 1-3 | -0.196 to -0.050 | 0.260-0.600 | >=0.600 |

Batch 4 was not tested separately because only five retained samples were available.

**B. Association between acquisition batch and NMF subtype**

| **Analysis** | **Estimate** | ***P* value** |
| --- | --- | --- |
| Fisher's exact test | - | 0.547 |
| Cramer's V | 0.126 | - |

**C. Sequential PERMANOVA**

| **Matrix** | **Term** | **R2** | ***P* value** |
| --- | --- | --- | --- |
| Original | NMF subtype | 0.1160 | <0.001 |
| Original | Acquisition batch | 0.0375 | <0.001 |
| Batch-adjusted | NMF subtype | 0.1158 | <0.001 |
| Batch-adjusted | Acquisition batch | 0.0021 | 1.000 |

Subtype was entered before acquisition batch in the sequential PERMANOVA.

**D. Batch-adjusted NMF sensitivity analysis**

| **Metric** | **Original k=3** | **Batch-adjusted k=3** |
| --- | --- | --- |
| NMF runs | 100 | 100 |
| Cophenetic correlation | 0.9797 | 0.9677 |
| Mean consensus silhouette | 0.8965 | 0.8137 |
| Adjusted Rand index versus original | - | 0.520 |
| Overall membership agreement | - | 79.2% |
| S1 membership retention | - | 13/26 (50.0%) |
| S2 membership retention | - | 31/33 (93.9%) |
| S3 membership retention | - | 32/37 (86.5%) |

The batch-adjusted NMF analysis used an intercept-only batch-adjustment model without retaining the original subtype labels.

|  |  |  |
| --- | --- | --- |
|  |  |  |
|  |  |  |
|  |  |  |
|  |  |  |
|  |  |  |

Supplementary Table S3. **Pearson and Spearman centroid correlations, S3-like margins, and ambiguous assignment rates in organ-dysfunction groups.**

| **Projection** | **Method** | **Clinical group** | **n** | **Comparator centroid r, median [IQR]** | **S3 centroid r, median [IQR]** | **S3-like margin, median [IQR]** | **Greater similarity to S3, n/N**  **(%)** | **Ambiguous assignment, n/N**  **(%)** |
| --- | --- | --- | --- | --- | --- | --- | --- | --- |
| S3 vs S1 | Pearson | S-AKI | 48 | 0.713 [0.672, 0.779] | 0.827 [0.779, 0.869] | 0.114 [0.009, 0.177] | 37/48 (77.1%) | 10/48 (20.8%) |
| S3 vs S1 | Pearson | S-ARDS | 46 | 0.721 [0.685, 0.764] | 0.851 [0.805, 0.872] | 0.121 [0.059, 0.175] | 39/46 (84.8%) | 4/46 (8.7%) |
| S3 vs S1 | Pearson | S-ARDS&AKI | 41 | 0.708 [0.663, 0.755] | 0.785 [0.755, 0.840] | 0.074 [0.013, 0.155] | 31/41 (75.6%) | 8/41 (19.5%) |
| S3 vs S1 | Spearman | S-AKI | 48 | 0.684 [0.623, 0.742] | 0.769 [0.703, 0.795] | 0.077 [-0.034, 0.152] | 32/48 (66.7%) | 11/48 (22.9%) |
| S3 vs S1 | Spearman | S-ARDS | 46 | 0.665 [0.623, 0.719] | 0.768 [0.739, 0.790] | 0.094 [0.024, 0.168] | 37/46 (80.4%) | 10/46 (21.7%) |
| S3 vs S1 | Spearman | S-ARDS&AKI | 41 | 0.662 [0.611, 0.712] | 0.705 [0.667, 0.756] | 0.058 [-0.025, 0.143] | 26/41 (63.4%) | 9/41 (22.0%) |
| S3 vs S2 | Pearson | S-AKI | 48 | 0.786 [0.751, 0.821] | 0.795 [0.770, 0.819] | 0.007 [-0.025, 0.032] | 28/48 (58.3%) | 38/48 (79.2%) |
| S3 vs S2 | Pearson | S-ARDS | 46 | 0.774 [0.741, 0.794] | 0.798 [0.772, 0.819] | 0.032 [-0.008, 0.063] | 33/46 (71.7%) | 26/46 (56.5%) |
| S3 vs S2 | Pearson | S-ARDS&AKI | 41 | 0.745 [0.711, 0.780] | 0.761 [0.707, 0.795] | 0.006 [-0.038, 0.060] | 22/41 (53.7%) | 22/41 (53.7%) |
| S3 vs S2 | Spearman | S-AKI | 48 | 0.715 [0.654, 0.765] | 0.707 [0.671, 0.738] | -0.010 [-0.047, 0.021] | 22/48 (45.8%) | 31/48 (64.6%) |
| S3 vs S2 | Spearman | S-ARDS | 46 | 0.667 [0.621, 0.736] | 0.703 [0.656, 0.730] | 0.030 [-0.027, 0.064] | 31/46 (67.4%) | 21/46 (45.7%) |
| S3 vs S2 | Spearman | S-ARDS&AKI | 41 | 0.654 [0.592, 0.716] | 0.649 [0.615, 0.697] | -0.001 [-0.056, 0.064] | 20/41 (48.8%) | 15/41 (36.6%) |

Values are median [IQR]. The S3-like margin was calculated as r(S3 centroid) - r(comparator centroid); positive values indicate greater similarity to S3. Ambiguous assignment was operationally defined as |margin| < 0.05. Nearest-centroid assignments represent relative molecular similarity, not definitive subtype membership. IQR, interquartile range; S-AKI, sepsis-associated acute kidney injury; S-ARDS, sepsis-associated acute respiratory distress syndrome; S-ARDS&AKI, concurrent S-ARDS and S-AKI.

Supplementary Table S4. Multivariable and sensitivity analyses of S3-like proteomic similarity

| **Projection space** | **Comparison** | **Adjustment model** | **Model N** | **Adjusted β**  **(95% CI)** | **P value** | **BH q** |
| --- | --- | --- | --- | --- | --- | --- |
| **Panel A. Primary and organ-support-adjusted analyses** | | | | | | |
| S3 vs S1 | S-AKI vs S1 | APACHE II + septic shock + comorbidity burden | 161 | 1.251 (0.809, 1.693) | <0.001 | <0.001 |
| S3 vs S1 | S-AKI vs S1 | Base model + mechanical ventilation + CRRT | 157 | 1.224 (0.763, 1.685) | <0.001 | <0.001 |
| S3 vs S1 | S-ARDS vs S1 | APACHE II + septic shock + comorbidity burden | 161 | 1.465 (1.069, 1.861) | <0.001 | <0.001 |
| S3 vs S1 | S-ARDS vs S1 | Base model + mechanical ventilation + CRRT | 157 | 1.391 (0.966, 1.815) | <0.001 | <0.001 |
| S3 vs S1 | S-ARDS&AKI vs S1 | APACHE II + septic shock + comorbidity burden | 161 | 1.210 (0.789, 1.630) | <0.001 | <0.001 |
| S3 vs S1 | S-ARDS&AKI vs S1 | Base model + mechanical ventilation + CRRT | 157 | 1.043 (0.556, 1.530) | <0.001 | <0.001 |
| S3 vs S2 | S-AKI vs S2 | APACHE II + septic shock + comorbidity burden | 168 | 0.874 (0.482, 1.265) | <0.001 | <0.001 |
| S3 vs S2 | S-AKI vs S2 | Base model + mechanical ventilation + CRRT | 163 | 0.877 (0.471, 1.282) | <0.001 | <0.001 |
| S3 vs S2 | S-ARDS vs S2 | APACHE II + septic shock + comorbidity burden | 168 | 1.409 (1.038, 1.780) | <0.001 | <0.001 |
| S3 vs S2 | S-ARDS vs S2 | Base model + mechanical ventilation + CRRT | 163 | 1.393 (0.995, 1.790) | <0.001 | <0.001 |
| S3 vs S2 | S-ARDS&AKI vs S2 | APACHE II + septic shock + comorbidity burden | 168 | 0.940 (0.492, 1.387) | <0.001 | <0.001 |
| S3 vs S2 | S-ARDS&AKI vs S2 | Base model + mechanical ventilation + CRRT | 163 | 0.835 (0.275, 1.395) | 0.004 | 0.004 |
| **Panel B. Alternative severity and shock adjustment models** | | | | | | |
| S3 vs S1 | S-AKI vs S1 | APACHE II + vasopressor use + mechanical ventilation + CRRT + comorbidity burden | 157 | 1.276 (0.792, 1.759) | <0.001 | — |
| S3 vs S1 | S-AKI vs S1 | SOFA + septic shock + comorbidity burden | 161 | 1.309 (0.863, 1.755) | <0.001 | — |
| S3 vs S1 | S-AKI vs S1 | SOFA + vasopressor use + mechanical ventilation + CRRT + comorbidity burden | 157 | 1.355 (0.863, 1.847) | <0.001 | — |
| S3 vs S1 | S-ARDS vs S1 | APACHE II + vasopressor use + mechanical ventilation + CRRT + comorbidity burden | 157 | 1.413 (0.988, 1.837) | <0.001 | — |
| S3 vs S1 | S-ARDS vs S1 | SOFA + septic shock + comorbidity burden | 161 | 1.501 (1.111, 1.890) | <0.001 | — |
| S3 vs S1 | S-ARDS vs S1 | SOFA + vasopressor use + mechanical ventilation + CRRT + comorbidity burden | 157 | 1.455 (1.027, 1.882) | <0.001 | — |
| S3 vs S1 | S-ARDS&AKI vs S1 | APACHE II + vasopressor use + mechanical ventilation + CRRT + comorbidity burden | 157 | 1.104 (0.617, 1.592) | <0.001 | — |
| S3 vs S1 | S-ARDS&AKI vs S1 | SOFA + septic shock + comorbidity burden | 161 | 1.265 (0.803, 1.727) | <0.001 | — |
| S3 vs S1 | S-ARDS&AKI vs S1 | SOFA + vasopressor use + mechanical ventilation + CRRT + comorbidity burden | 157 | 1.169 (0.659, 1.680) | <0.001 | — |
| S3 vs S2 | S-AKI vs S2 | APACHE II + vasopressor use + mechanical ventilation + CRRT + comorbidity burden | 163 | 0.883 (0.467, 1.300) | <0.001 | — |
| S3 vs S2 | S-AKI vs S2 | SOFA + septic shock + comorbidity burden | 168 | 0.945 (0.533, 1.358) | <0.001 | — |
| S3 vs S2 | S-AKI vs S2 | SOFA + vasopressor use + mechanical ventilation + CRRT + comorbidity burden | 163 | 0.951 (0.520, 1.383) | <0.001 | — |
| S3 vs S2 | S-ARDS vs S2 | APACHE II + vasopressor use + mechanical ventilation + CRRT + comorbidity burden | 163 | 1.388 (0.990, 1.787) | <0.001 | — |
| S3 vs S2 | S-ARDS vs S2 | SOFA + septic shock + comorbidity burden | 168 | 1.413 (1.044, 1.783) | <0.001 | — |
| S3 vs S2 | S-ARDS vs S2 | SOFA + vasopressor use + mechanical ventilation + CRRT + comorbidity burden | 163 | 1.350 (0.946, 1.754) | <0.001 | — |
| S3 vs S2 | S-ARDS&AKI vs S2 | APACHE II + vasopressor use + mechanical ventilation + CRRT + comorbidity burden | 163 | 0.839 (0.266, 1.412) | 0.004 | — |
| S3 vs S2 | S-ARDS&AKI vs S2 | SOFA + septic shock + comorbidity burden | 168 | 1.079 (0.607, 1.551) | <0.001 | — |
| S3 vs S2 | S-ARDS&AKI vs S2 | SOFA + vasopressor use + mechanical ventilation + CRRT + comorbidity burden | 163 | 0.917 (0.357, 1.477) | 0.001 | — |
| **Panel C. Spearman-margin sensitivity analyses** | | | | | | |
| S3 vs S1 | S-AKI vs S1 | APACHE II + septic shock + comorbidity burden | 161 | 1.238 (0.839, 1.637) | <0.001 | <0.001 |
| S3 vs S1 | S-AKI vs S1 | Base model + mechanical ventilation + CRRT | 157 | 1.211 (0.804, 1.618) | <0.001 | <0.001 |
| S3 vs S1 | S-ARDS vs S1 | APACHE II + septic shock + comorbidity burden | 161 | 1.483 (1.128, 1.838) | <0.001 | <0.001 |
| S3 vs S1 | S-ARDS vs S1 | Base model + mechanical ventilation + CRRT | 157 | 1.427 (1.047, 1.807) | <0.001 | <0.001 |
| S3 vs S1 | S-ARDS&AKI vs S1 | APACHE II + septic shock + comorbidity burden | 161 | 1.132 (0.738, 1.526) | <0.001 | <0.001 |
| S3 vs S1 | S-ARDS&AKI vs S1 | Base model + mechanical ventilation + CRRT | 157 | 0.975 (0.507, 1.444) | <0.001 | <0.001 |
| S3 vs S2 | S-AKI vs S2 | APACHE II + septic shock + comorbidity burden | 168 | 0.806 (0.418, 1.193) | <0.001 | <0.001 |
| S3 vs S2 | S-AKI vs S2 | Base model + mechanical ventilation + CRRT | 163 | 0.815 (0.409, 1.220) | <0.001 | <0.001 |
| S3 vs S2 | S-ARDS vs S2 | APACHE II + septic shock + comorbidity burden | 168 | 1.391 (1.018, 1.763) | <0.001 | <0.001 |
| S3 vs S2 | S-ARDS vs S2 | Base model + mechanical ventilation + CRRT | 163 | 1.401 (0.999, 1.803) | <0.001 | <0.001 |
| S3 vs S2 | S-ARDS&AKI vs S2 | APACHE II + septic shock + comorbidity burden | 168 | 0.980 (0.535, 1.425) | <0.001 | <0.001 |
| S3 vs S2 | S-ARDS&AKI vs S2 | Base model + mechanical ventilation + CRRT | 163 | 0.916 (0.347, 1.485) | 0.002 | 0.002 |
| **Panel D. Sensitivity analysis using all Sepsis-only patients as the reference population** | | | | | | |
| S3 vs S1 | S-AKI vs all Sepsis-only | APACHE II + septic shock + comorbidity burden | 231 | 0.109 (-0.227, 0.445) | 0.523 | 0.829 |
| S3 vs S1 | S-AKI vs all Sepsis-only | Base model + mechanical ventilation + CRRT | 225 | 0.078 (-0.277, 0.433) | 0.667 | 0.829 |
| S3 vs S1 | S-ARDS vs all Sepsis-only | APACHE II + septic shock + comorbidity burden | 231 | 0.335 (0.047, 0.623) | 0.023 | 0.068 |
| S3 vs S1 | S-ARDS vs all Sepsis-only | Base model + mechanical ventilation + CRRT | 225 | 0.271 (-0.057, 0.599) | 0.105 | 0.314 |
| S3 vs S1 | S-ARDS&AKI vs all Sepsis-only | APACHE II + septic shock + comorbidity burden | 231 | 0.057 (-0.253, 0.368) | 0.717 | 0.829 |
| S3 vs S1 | S-ARDS&AKI vs all Sepsis-only | Base model + mechanical ventilation + CRRT | 225 | -0.084 (-0.501, 0.333) | 0.691 | 0.829 |
| S3 vs S2 | S-AKI vs all Sepsis-only | APACHE II + septic shock + comorbidity burden | 231 | 0.039 (-0.317, 0.395) | 0.829 | 0.829 |
| S3 vs S2 | S-AKI vs all Sepsis-only | Base model + mechanical ventilation + CRRT | 225 | 0.093 (-0.282, 0.468) | 0.625 | 0.829 |
| S3 vs S2 | S-ARDS vs all Sepsis-only | APACHE II + septic shock + comorbidity burden | 231 | 0.576 (0.246, 0.906) | <0.001 | 0.004 |
| S3 vs S2 | S-ARDS vs all Sepsis-only | Base model + mechanical ventilation + CRRT | 225 | 0.540 (0.209, 0.870) | 0.001 | 0.009 |
| S3 vs S2 | S-ARDS&AKI vs all Sepsis-only | APACHE II + septic shock + comorbidity burden | 231 | 0.068 (-0.342, 0.479) | 0.744 | 0.829 |
| S3 vs S2 | S-ARDS&AKI vs all Sepsis-only | Base model + mechanical ventilation + CRRT | 225 | -0.022 (-0.516, 0.473) | 0.931 | 0.931 |
| **Panel E. Single-organ sensitivity analyses excluding S-ARDS&AKI** | | | | | | |
| S3 vs S1 | S-AKI vs S1 | APACHE II + septic shock + comorbidity burden | 120 | 1.219 (0.761, 1.677) | <0.001 | — |
| S3 vs S1 | S-AKI vs S1 | Base model + mechanical ventilation + CRRT | 116 | 1.233 (0.742, 1.725) | <0.001 | — |
| S3 vs S1 | S-ARDS vs S1 | APACHE II + septic shock + comorbidity burden | 120 | 1.437 (1.034, 1.841) | <0.001 | — |
| S3 vs S1 | S-ARDS vs S1 | Base model + mechanical ventilation + CRRT | 116 | 1.380 (0.944, 1.816) | <0.001 | — |
| S3 vs S2 | S-AKI vs S2 | APACHE II + septic shock + comorbidity burden | 127 | 0.840 (0.450, 1.231) | <0.001 | — |
| S3 vs S2 | S-AKI vs S2 | Base model + mechanical ventilation + CRRT | 122 | 0.877 (0.463, 1.290) | <0.001 | — |
| S3 vs S2 | S-ARDS vs S2 | APACHE II + septic shock + comorbidity burden | 127 | 1.394 (1.019, 1.770) | <0.001 | — |
| S3 vs S2 | S-ARDS vs S2 | Base model + mechanical ventilation + CRRT | 122 | 1.364 (0.959, 1.769) | <0.001 | — |

Adjusted β values represent standardized differences in S3-like similarity margins. The base model included APACHE II score, septic shock, and chronic-comorbidity burden; the organ-support model additionally included mechanical ventilation and CRRT. P values were adjusted by the Benjamini–Hochberg method within prespecified model families. A dash indicates that no BH-adjusted q value was prespecified for that secondary sensitivity analysis. CRRT, continuous renal replacement therapy.

Supplementary Table S5. **Sensitivity analysis using k-nearest-neighbor imputation**

| **Analysis** | **Comparison or assessment** | **Primary analysis** | **KNN sensitivity analysis** | **Agreement with the primary analysis** |
| --- | --- | --- | --- | --- |
| NMF subtype assignment | Sepsis-only cohort (n = 96) | S1, n = 26; S2, n = 33; S3, n = 37 | 86 of 96 assignments concordant | Concordance, 89.6%; adjusted Rand index, 0.707 |
| Differential expression | S3 versus S2 | 1,440 significant proteins | 569 significant proteins | 548 overlapping proteins; retention, 38.1%; log2FC rho = 0.608; directional concordance, 83.0% |
| Differential expression | Sepsis&Complications versus S2 | 852 significant proteins | 295 significant proteins | 276 overlapping proteins; retention, 32.4%; log2FC rho = 0.714; directional concordance, 83.7% |
| Candidate-protein assessment | Fixed set of 36 shared candidates | 36 candidates | 36 retained the expected direction; 31 met the original criteria in both comparisons | Directional concordance, 100.0%; strict-criterion retention, 86.1% |
| Panel assessment | Fixed 15-protein panel | 15 proteins | 15 retained the expected direction; 13 met the original criteria in both comparisons | Directional concordance, 100.0%; strict-criterion retention, 86.7% |

In the primary analysis, remaining missing values were imputed separately for each sample using a left-shifted Gaussian distribution (shift, 1.8 standard deviations; width, 0.3 standard deviations; random seed, 2026). For sensitivity analysis, missing values in the same globally filtered, log2-transformed, median-normalized matrix were alternatively imputed using KNN (k = 10; random seed, 2026). The same samples, differential expression model, significance criteria, NMF rank, and number of NMF runs were used. differential expression significance was defined as Benjamini-Hochberg-adjusted P < 0.05 and |log2 fold change| > log2(1.5). Retention denotes the proportion of proteins significant in the primary analysis that remained significant after KNN imputation. Directional concordance for the differential expression analyses was calculated across all tested proteins. KNN, k-nearest neighbor; NMF, non-negative matrix factorization.

**Supplementary Table S6. Performance, calibration, threshold stability, decision-curve analysis, and optimism-corrected estimates from repeated nested internal validation.**

| **Domain** | **Measure** | **Sepsis&Complications vs S2** | **S3 vs S2** |
| --- | --- | --- | --- |
| Validation cohort | Comparison | Sepsis&Complications vs S2 | S3 vs S2 |
| Validation cohort | Groups included | S-AKI, S-ARDS, and concurrent S-ARDS&AKI vs S2 | S3 vs S2 |
| Validation cohort | Sample size, positive/negative | 168 (135/33) | 70 (37/33) |
| Validation cohort | Positive-class prevalence | 0.804 | 0.529 |
| Validation design | Repeated outer validation | 100 repeats; 500 folds | 100 repeats; 500 folds |
| Validation design | Successful outer folds | 500/500 | 500/500 |
| Discrimination | AUROC, 95% CI | 0.847 (0.777-0.902) | 0.956 (0.912-0.989) |
| Discrimination | PR-AUC, 95% CI | 0.955 (0.928-0.976) | 0.951 (0.904-0.989) |
| Repeat-level calibration | Calibration intercept, 95% CI | 0.002 (-0.127-0.129) | -0.142 (-0.466-0.211) |
| Repeat-level calibration | Calibration slope, 95% CI | 1.531 (0.976-2.384) | 1.865 (1.401-44.244) |
| Repeat-level calibration | Brier score, 95% CI | 0.127 (0.114-0.143) | 0.082 (0.052-0.115) |
| Decision threshold | Median threshold; IQR | 0.784; IQR 0.768-0.798 | 0.557; IQR 0.502-0.618 |
| Decision threshold | 2.5th-97.5th percentiles | 0.733-0.822 | 0.424-0.742 |
| Patient-level consensus classification | Confusion matrix counts | TN 29; FP 4; FN 31; TP 104 | TN 29; FP 4; FN 2; TP 35 |
| Patient-level consensus classification | Sensitivity | 0.770 | 0.946 |
| Patient-level consensus classification | Specificity | 0.879 | 0.879 |
| Patient-level consensus classification | Positive predictive value | 0.963 | 0.897 |
| Patient-level consensus classification | Negative predictive value | 0.483 | 0.935 |
| Patient-level consensus classification | F1 score | 0.856 | 0.921 |
| Bootstrap optimism correction | Apparent AUROC | 0.918 | 0.993 |
| Bootstrap optimism correction | Mean AUROC optimism | 0.059 | 0.013 |
| Bootstrap optimism correction | Optimism-corrected AUROC | 0.859 (0.808-0.912) | 0.980 (0.956-0.993) |
| Bootstrap optimism correction | Apparent PR-AUC | 0.978 | 0.994 |
| Bootstrap optimism correction | Mean PR-AUC optimism | 0.018 | 0.015 |
| Bootstrap optimism correction | Optimism-corrected PR-AUC | 0.960 (0.940-0.977) | 0.979 (0.945-0.994) |
| Patient-aggregated calibration | Raw nested-workflow OOF: intercept; slope; Brier score | 0.002; 1.725; 0.125 | -0.135; 2.096; 0.078 |
| Patient-aggregated calibration | Within-fold recalibrated nested-workflow OOF: intercept; slope; Brier score | -0.004; 0.999; 0.120 | -0.122; 1.018; 0.068 |
| Decision-curve analysis | Threshold range with net benefit greater than treat-all and treat-none | 0.44-0.95 | Not assessed |
| Supportive fixed 15-protein model | AUROC, 95% CI | 0.887 (0.824-0.940) | 0.961 (0.899-0.998) |
| Supportive fixed 15-protein model | PR-AUC, 95% CI | 0.967 (0.943-0.986) | 0.930 (0.826-0.998) |
| Supportive fixed 15-protein model | Raw patient-aggregated OOF: intercept; slope; Brier score | 0.005; 2.011; 0.113 | -0.021; 2.200; 0.081 |
| Supportive fixed 15-protein model | Within-fold recalibrated patient-aggregated OOF: intercept; slope; Brier score | 0.011; 0.994; 0.101 | 0.022; 0.898; 0.060 |
| Supportive fixed 15-protein model | DCA threshold range with net benefit greater than treat-all and treat-none | 0.17-0.36; 0.38-0.95 | Not assessed |

AUROC, area under the receiver operating characteristic curve; PR-AUC, area under the precision-recall curve; OOF, out-of-fold; IQR, interquartile range; DCA, decision-curve analysis; TN, true negative; FP, false positive; FN, false negative; TP, true positive. Patient-aggregated measures were calculated from repeated OOF probabilities. The fixed 15-protein model analysis was supportive because the panel was derived in the same cohort.

**Supplementary Table S7.** **Shared-candidate and task-specific coefficient stability of the originally reported 15-protein panel across repeated nested resampling.**

| **Protein** | **Shared candidate stability** | | | **Primary model: Sepsis&Complications vs S2** | | **Secondary model: S3 vs S2** | | |
| --- | --- | --- | --- | --- | --- | --- | --- | --- |
|  | **Candidate frequency, n/N (%)** | **Mean log2FC, S3 vs S2** | **Mean log2FC, Sepsis&Complications vs S2** | **Non-zero coefficient frequency, n/N (%)** | **Median coefficient [IQR]** | **Non-zero coefficient frequency, n/N (%)** | **Positive coefficient among selected folds, n/N (%)** | **Median coefficient [IQR]** |
| RNASE1 | 500/500 (100.0%) | 1.022 | 1.314 | 500/500 (100.0%) | 0.204 [0.143, 0.314] | 462/500 (92.4%) | 462/462 (100.0%) | 0.132 [0.096, 0.180] |
| OIT3 | 497/500 (99.4%) | 0.937 | 1.098 | 495/500 (99.0%) | 0.145 [0.105, 0.226] | 423/500 (84.6%) | 423/423 (100.0%) | 0.108 [0.066, 0.169] |
| IGHV3-38 | 498/500 (99.6%) | 0.969 | 0.760 | 496/500 (99.2%) | 0.138 [0.096, 0.192] | 481/500 (96.2%) | 481/481 (100.0%) | 0.161 [0.123, 0.214] |
| RBP4 | 500/500 (100.0%) | 0.951 | 1.051 | 497/500 (99.4%) | 0.125 [0.091, 0.178] | 417/500 (83.4%) | 417/417 (100.0%) | 0.110 [0.078, 0.161] |
| IGFBP4 | 294/500 (58.8%) | 0.625 | 0.969 | 281/500 (56.2%) | 0.119 [0.083, 0.156] | 74/500 (14.8%) | 74/74 (100.0%) | 0.026 [0.011, 0.044] |
| CFD | 498/500 (99.6%) | 0.750 | 0.757 | 450/500 (90.0%) | 0.076 [0.056, 0.102] | 472/500 (94.4%) | 472/472 (100.0%) | 0.162 [0.119, 0.215] |
| IGLV1-40 | 456/500 (91.2%) | 0.888 | 0.763 | 416/500 (83.2%) | 0.071 [0.041, 0.097] | 227/500 (45.4%) | 227/227 (100.0%) | 0.050 [0.023, 0.080] |
| KRTDAP | 479/500 (95.8%) | 1.104 | 0.854 | 441/500 (88.2%) | 0.078 [0.051, 0.114] | 420/500 (84.0%) | 420/420 (100.0%) | 0.113 [0.075, 0.166] |
| ATRN | 484/500 (96.8%) | 1.079 | 0.685 | 379/500 (75.8%) | 0.044 [0.032, 0.062] | 386/500 (77.2%) | 386/386 (100.0%) | 0.148 [0.112, 0.186] |
| CST3 | 389/500 (77.8%) | 0.644 | 0.812 | 325/500 (65.0%) | 0.072 [0.055, 0.092] | 131/500 (26.2%) | 131/131 (100.0%) | 0.027 [0.011, 0.050] |
| IGLV1-36 | 486/500 (97.2%) | 2.499 | 2.038 | 389/500 (77.8%) | 0.058 [0.033, 0.087] | 59/500 (11.8%) | 48/59 (81.4%) | 0.015 [0.002, 0.042] |
| IGHV1-18 | 498/500 (99.6%) | 1.620 | 0.951 | 336/500 (67.2%) | 0.035 [0.021, 0.061] | 401/500 (80.2%) | 401/401 (100.0%) | 0.103 [0.069, 0.144] |
| APOH | 486/500 (97.2%) | 0.945 | 0.705 | 348/500 (69.6%) | 0.042 [0.027, 0.058] | 341/500 (68.2%) | 341/341 (100.0%) | 0.087 [0.058, 0.117] |
| ACKR1 | 121/500 (24.2%) | 1.124 | 1.056 | 101/500 (20.2%) | 0.063 [0.038, 0.098] | 31/500 (6.2%) | 31/31 (100.0%) | 0.016 [0.008, 0.038] |
| CCL14 | 490/500 (98.0%) | 0.811 | 0.808 | 354/500 (70.8%) | 0.049 [0.031, 0.077] | 167/500 (33.4%) | 167/167 (100.0%) | 0.028 [0.015, 0.048] |

Candidate frequency reflected concordant upregulation in both training-fold comparisons. The primary task was Sepsis&Complications versus S2, and the secondary task was S3 versus S2. All selected primary-task coefficients were positive. Coefficients refer to standardized predictors. NMF subtype labels were fixed before resampling.

Supplementary Table S8. Detection rates of representative co-isolate indicators across S1, S2, and S3 in the original non-imputed proteomic matrix.

| **Category** | **Protein** | **S1, n/N (%)** | **S2, n/N (%)** | **S3, n/N (%)** |
| --- | --- | --- | --- | --- |
| Albumin/apolipoprotein and lipoprotein-related | ALB | 26/26 (100.0%) | 33/33 (100.0%) | 37/37 (100.0%) |
| Albumin/apolipoprotein and lipoprotein-related | APOA1 | 26/26 (100.0%) | 33/33 (100.0%) | 37/37 (100.0%) |
| Albumin/apolipoprotein and lipoprotein-related | APOA2 | 26/26 (100.0%) | 33/33 (100.0%) | 37/37 (100.0%) |
| Albumin/apolipoprotein and lipoprotein-related | APOB | 26/26 (100.0%) | 33/33 (100.0%) | 37/37 (100.0%) |
| Albumin/apolipoprotein and lipoprotein-related | APOC1 | 26/26 (100.0%) | 33/33 (100.0%) | 37/37 (100.0%) |
| Albumin/apolipoprotein and lipoprotein-related | APOC2 | 26/26 (100.0%) | 33/33 (100.0%) | 37/37 (100.0%) |
| Albumin/apolipoprotein and lipoprotein-related | APOC3 | 26/26 (100.0%) | 33/33 (100.0%) | 37/37 (100.0%) |
| Albumin/apolipoprotein and lipoprotein-related | APOC4 | 26/26 (100.0%) | 33/33 (100.0%) | 37/37 (100.0%) |
| Albumin/apolipoprotein and lipoprotein-related | APOE | 26/26 (100.0%) | 33/33 (100.0%) | 37/37 (100.0%) |
| Albumin/apolipoprotein and lipoprotein-related | LPA | 26/26 (100.0%) | 33/33 (100.0%) | 37/37 (100.0%) |
| Intracellular-compartment-associated | CANX | 26/26 (100.0%) | 33/33 (100.0%) | 37/37 (100.0%) |
| Intracellular-compartment-associated | HSP90B1 | 26/26 (100.0%) | 33/33 (100.0%) | 37/37 (100.0%) |
| Intracellular-compartment-associated | HSPA5 | 26/26 (100.0%) | 33/33 (100.0%) | 37/37 (100.0%) |
| Intracellular-compartment-associated | GOLGA2 | 0/26 (0.0%) | 0/33 (0.0%) | 0/37 (0.0%) |
| Intracellular-compartment-associated | TOMM20 | 3/26 (11.5%) | 3/33 (9.1%) | 2/37 (5.4%) |
| Intracellular-compartment-associated | VDAC1 | 26/26 (100.0%) | 33/33 (100.0%) | 37/37 (100.0%) |
| Intracellular-compartment-associated | VDAC2 | 26/26 (100.0%) | 33/33 (100.0%) | 36/37 (97.3%) |
| Intracellular-compartment-associated | CYC1 | 20/26 (76.9%) | 30/33 (90.9%) | 25/37 (67.6%) |

Detection rates were calculated from the original non-imputed proteomic matrix and are presented as n/N (%). S1, n = 26; S2, n = 33; S3, n = 37. Indicators were selected with reference to the MISEV protein-characterization framework and were evaluated as representative co-isolate or intracellular-compartment-associated indicators rather than universal negative EV markers.

Supplementary Table S9. S3 vs S2 comparison of representative co-isolate indicators.

| **Category** | **Protein** | **S2 median [IQR]** | **S3 median [IQR]** | **Median difference**  **(S3−S2)** | **Wilcoxon P value** | **BH-adjusted P value** |
| --- | --- | --- | --- | --- | --- | --- |
| Albumin/apolipoprotein and lipoprotein-related | **ALB** | 13.64 [13.47–13.93] | 14.19 [13.78–14.40] | +0.543 | <0.001 | <0.001 |
| Albumin/apolipoprotein and lipoprotein-related | APOA1 | 14.30 [14.02–14.54] | 14.44 [14.19–14.61] | +0.146 | 0.169 | 0.270 |
| Albumin/apolipoprotein and lipoprotein-related | APOA2 | 12.78 [11.95–13.27] | 12.61 [12.21–13.20] | -0.173 | 0.981 | 0.981 |
| Albumin/apolipoprotein and lipoprotein-related | APOB | 13.22 [12.71–13.76] | 13.23 [12.76–13.71] | +0.002 | 0.953 | 0.981 |
| Albumin/apolipoprotein and lipoprotein-related | APOC1 | 12.70 [12.25–13.20] | 12.46 [11.17–12.90] | -0.242 | 0.090 | 0.173 |
| Albumin/apolipoprotein and lipoprotein-related | APOC2 | 14.79 [14.44–15.30] | 14.36 [14.05–14.89] | -0.438 | 0.036 | 0.116 |
| Albumin/apolipoprotein and lipoprotein-related | APOC3 | 13.73 [12.72–14.08] | 13.40 [12.57–13.99] | -0.325 | 0.353 | 0.513 |
| Albumin/apolipoprotein and lipoprotein-related | APOC4 | 11.72 [10.67–12.21] | 11.52 [10.41–12.27] | -0.196 | 0.769 | 0.920 |
| Albumin/apolipoprotein and lipoprotein-related | APOE | 15.24 [14.51–15.61] | 15.02 [14.78–15.18] | -0.228 | 0.404 | 0.538 |
| Albumin/apolipoprotein and lipoprotein-related | LPA | 12.42 [10.76–12.81] | 12.16 [10.92–12.98] | -0.261 | 0.805 | 0.920 |
| Intracellular-compartment-associated | CANX | 9.20 [8.47–9.72] | 8.88 [8.21–9.20] | -0.329 | 0.095 | 0.173 |
| Intracellular-compartment-associated | HSP90B1 | 9.13 [8.77–9.76] | 8.84 [8.51–9.12] | -0.286 | 0.057 | 0.151 |
| Intracellular-compartment-associated | HSPA5 | 9.72 [9.10–10.11] | 9.36 [8.81–9.60] | -0.365 | 0.026 | 0.105 |
| Intracellular-compartment-associated | VDAC1 | 7.31 [7.05–7.60] | 7.13 [6.11–7.43] | -0.184 | 0.097 | 0.173 |
| Intracellular-compartment-associated | VDAC2 | 6.93 [6.41–7.35] | 6.23 [5.79–6.64] | -0.702 | <0.001 | <0.001 |
| Intracellular-compartment-associated | CYC1 | 6.20 [5.37–6.63] | 5.40 [4.04–5.91] | -0.800 | 0.003 | 0.014 |

Values are log2-transformed, median-normalized intensities. IQR, interquartile range; BH, Benjamini–Hochberg.S3 and S2 were compared using the Wilcoxon rank-sum test. Median differences were calculated as S3 minus S2, and P values were adjusted across all evaluated markers using the BH method
